# Pleiotropic Effects of Variants in Dementia Genes in Parkinson Disease

**DOI:** 10.3389/fnins.2018.00230

**Published:** 2018-04-10

**Authors:** Laura Ibanez, Umber Dube, Albert A. Davis, Maria V. Fernandez, John Budde, Breanna Cooper, Monica Diez-Fairen, Sara Ortega-Cubero, Pau Pastor, Joel S. Perlmutter, Carlos Cruchaga, Bruno A. Benitez

**Affiliations:** ^1^Department of Psychiatry, Washington University, Saint Louis, MO, United States; ^2^Department of Neurology, Washington University, Saint Louis, MO, United States; ^3^Centro de Investigación Biomédica en Red de Enfermedades Neurodegenerativas, Instituto de Salud Carlos III, Madrid, Spain; ^4^Movement Disorders Unit, Department of Neurology, University Hospital Mutua de Terrassa, Fundació per la Recerca Biomèdica i Social Mútua Terrassa, Terrassa, Barcelona, Spain; ^5^Department of Neurology and Neurosurgery, Hospital Universitario de Burgos, Burgos, Spain; ^6^Departments of Radiology, Neuroscience, Physical Therapy, and Occupational Therapy, Washington University, Saint Louis, MO, United States; ^7^Department of Medicine, Washington University, Saint Louis, MO, United States

**Keywords:** Parkinson disease, dementia, cognitive impairment, rare variants, *APP*, *PSEN1*, *PSEN2*, *GRN*

## Abstract

**Background:** The prevalence of dementia in Parkinson disease (PD) increases dramatically with advancing age, approaching 80% in patients who survive 20 years with the disease. Increasing evidence suggests clinical, pathological and genetic overlap between Alzheimer disease, dementia with Lewy bodies and frontotemporal dementia with PD. However, the contribution of the dementia-causing genes to PD risk, cognitive impairment and dementia in PD is not fully established.

**Objective:** To assess the contribution of coding variants in Mendelian dementia-causing genes on the risk of developing PD and the effect on cognitive performance of PD patients.

**Methods:** We analyzed the coding regions of the amyloid-beta precursor protein (*APP*), Presenilin 1 and 2 (*PSEN1, PSEN2*), and Granulin (*GRN*) genes from 1,374 PD cases and 973 controls using pooled-DNA targeted sequence, human exome-chip and whole-exome sequencing (WES) data by single variant and gene base (SKAT-O and burden tests) analyses. Global cognitive function was assessed using the Mini-Mental State Examination (MMSE) or the Montreal Cognitive Assessment (MoCA). The effect of coding variants in dementia-causing genes on cognitive performance was tested by multiple regression analysis adjusting for gender, disease duration, age at dementia assessment, study site and *APOE* carrier status.

**Results:** Known AD pathogenic mutations in the *PSEN1* (p.A79V) and *PSEN2* (p.V148I) genes were found in 0.3% of all PD patients. There was a significant burden of rare, likely damaging variants in the *GRN* and *PSEN1* genes in PD patients when compared with frequencies in the European population from the ExAC database. Multiple regression analysis revealed that PD patients carrying rare variants in the *APP, PSEN1, PSEN2*, and *GRN* genes exhibit lower cognitive tests scores than non-carrier PD patients (*p* = 2.0 × 10^−4^), independent of age at PD diagnosis, age at evaluation, *APOE* status or recruitment site.

**Conclusions:** Pathogenic mutations in the Alzheimer disease-causing genes (*PSEN1* and *PSEN2)* are found in sporadic PD patients. PD patients with cognitive decline carry rare variants in dementia-causing genes. Variants in genes causing Mendelian neurodegenerative diseases exhibit pleiotropic effects.

## Introduction

Recent genome-wide association studies (GWAS) have reported an overlap between Parkinson disease (PD) and the most common forms of dementia including Alzheimer disease (AD), dementia with Lewy bodies (DLB) and Frontotemporal dementia (FTD) (Guerreiro et al., 2015; Ferrari et al., 2017). Multiple variants associated with PD risk have also been identified as risk factors for AD, DLB, or FTD including variants in the following genes: *Triggering receptor expressed on myeloid cells 2 (TREM2), Microtubule-associated protein tau* (*MAPT), C9orf72, Glucocerebrosidase* (*GBA)*, and *Apolipoprotein E (APOE)* (Parsian et al., 2002; Harms et al., 2013; Davis et al., 2015; Benitez et al., 2016).

AD is the most common form of dementia and is characterized pathologically by the accumulation of amyloid plaques and neurofibrillary tangles. *Amyloid protein precursor* (*APP*), *presenilin-1* (*PSEN1*), and *presenilin-2* (*PSEN2*) mutations cause autosomal dominant forms of early-onset AD (Cruts et al., 2012). Approximately 220 pathogenic mutations in the *PSEN1* gene have been reported in AD patients worldwide, whereas 27 and 16 pathogenic mutations have been described in the *APP* and *PSEN2* genes, respectively (Cruts et al., 2012). Rare functional variants in *APP* (Schulte et al., 2015) and pathogenic mutations in *PSEN1*, and *PSEN2* have been also reported in PD patients (Takao et al., 2002; Jimenez-Escrig et al., 2004; Puschmann et al., 2009; Niwa et al., 2013). We recently reported the presence of *leucine-rich repeat kinase 2* (*LRRK2)*, p.G2019S mutation in members of two multigenerational families with AD and a suggestive association of variants in the *PTEN-induced putative kinase 1* (*PINK1)* gene with AD (Fernández et al., 2017). These results suggest a genetic overlap between familial AD and PD.

There is also an overlap of neuropathology in the brains of AD and PD patients. Approximately 50–60% of the sporadic and familial AD patients with pathogenic mutations in *APP, PSEN1*, or *PSEN2* genes exhibit widespread α-synuclein pathology (Meeus et al., 2012a). Abnormal cortical amyloid-beta (Aβ) deposition is present in 60% of PD patients with dementia (Kotzbauer et al., 2012), the burden of Aβ plaques inversely correlates with cognitive status in PD cases with dementia (Irwin et al., 2013) and the progression of the dementia in PD correlates with Lewy body (LB) and cortical AD-type pathology (Compta et al., 2011). In addition, AD-like changes in cerebrospinal fluid (CSF) biomarkers (Aβ levels) have been reported in PD patients (Terrelonge et al., 2015). Taken together, these results suggest that abnormal APP processing and Aβ accumulation occurs in PD. Here, we evaluate the genetic variation of *APP, PSEN1* and *PSEN2* genes in PD patients.

FTD may cause up to 10% of all cases of dementia and is the second most common cause of early-onset dementia (<65 years of age) (Ratnavalli et al., 2002). The three most common genetic causes of FTD are mutations in the genes *MAPT* and *granulin* (*GRN*), and expansions of a hexanucleotide repeat (GGGGCC) in the *C9orf72* gene. *C9orf72* expansions cause 5–12% of all FTD and 10–35% of familial FTD. *MAPT* or *GRN* mutations are found in 1–10% of all FTD and 5–25% of familial FTD. *MAPT* variants associated with FTD also increase the risk of developing PD (Pastor et al., 2000; Benitez et al., 2016). *C9orf72* expansions have been found in PD patients in some (Baizabal-Carvallo and Jankovic, 2016) but not all studies (Harms et al., 2013). Parkinsonism precedes the cognitive and behavioral symptoms of FTD by several years in patients with mutations in *GRN* (Baizabal-Carvallo and Jankovic, 2016). Up to 41% of FTD patients with *GRN* mutations exhibit parkinsonism (Josephs et al., 2007). In addition, *GRN* pathogenic mutations have been reported in PD patients (Brouwers et al., 2007; Rovelet-Lecrux et al., 2008). Here, we explore the role of variants in the *GRN* gene in PD patients.

PD is the most common neurodegenerative movement disorder, affecting ~1–2% of people over 60 years of age (Wright Willis et al., 2010). Clinically, PD patients exhibit bradykinesia, rest tremor, rigidity, and disturbances in balance. PD is characterized neuropathologically by the presence of α-synuclein-positive neuronal inclusions, commonly referred to as LBs and Lewy neurites, as well as neuronal loss in the substantia nigra. Genetically, familial autosomal dominant PD is caused by highly penetrant mutations in the *alpha-synuclein (SNCA)* and *LRRK2* genes, whereas autosomal recessive PD is caused by mutations in the *PARK2/PARKIN, PINK-1*, and *PARK7/DJ-1* genes (Petrucci et al., 2014). Recently, a GWAS identified at least 41 loci associated at genome-wide significant level with disease risk in individuals of European ancestry (Chang et al., 2017). The most statistically significant signals associated with PD are common variants located close to *SNCA, MAPT*, and *GBA* genes (Chang et al., 2017).

Dementia is one of the most common non-motor symptoms in PD (Foltynie et al., 2004; Emre et al., 2007). The prevalence of dementia in PD patients at any stage of disease ranges from 22 to 48% (Foltynie et al., 2004; Emre et al., 2007). Several risk factors for dementia in PD include severity of parkinsonism, the presence of non-motor symptoms, older age, male sex and presence of cognitive symptoms at PD diagnosis (Aarsland and Kurz, 2010). Relatives of PD patients exhibit a higher risk of dementia than relatives of control subjects, (Rocca et al., 2007) supporting a role of genetic factors in the development of dementia in PD. However, the genetic contributors to dementia in PD have yet to be fully established. Longitudinal studies have shown that the *APOE* ε4 allele, the *H1* haplotype in *MAPT* and mutations in the *GBA* gene are associated with a more rapid cognitive decline in PD (Morley et al., 2012; Collins and Williams-Gray, 2016; Liu et al., 2016). In contrast, a cross-sectional study showed that PD carriers of the *LRRK2*,p.G2019S mutation exhibit lower rates of dementia (Srivatsal et al., 2015). However, it is not clear how frequently mutations in dementia-causing genes occur in PD patients or whether variants in these genes relate to dementia in PD patients.

Here, we performed a systematic screening of known dementia-causing genes (*APP, PSEN1, PSEN2*, or *GRN* genes) in 821 PD cases and 423 controls from North America in addition to 553 PD patients and 550 healthy controls from Spain.

## Materials and methods

### Subjects

Three cohorts were included in this study: the Washington University in Saint Louis (WUSTL) Movement Disorder Center (MO, USA), the Parkinson's Progression Markers Initiative (PPMI) consortium (www.ppmi-info.org) and the Movement Disorders Unit at the University of Navarra (UN), School of Medicine (Navarra, Spain). PD clinical diagnoses were based on UK Brain Bank criteria (Hughes et al., 1992). Written informed consent was obtained from all participants prior to their enrollment. The Washington University in Saint Louis Human Research Protection Office (approval number: 201107095) approved the study. Demographic characteristics of these three populations have been published previously (Weintraub et al., 2013; Davis et al., 2015; Benitez et al., 2016). *The WUSTL cohort* included 490 non-hispanic white (NHW) PD cases [64% males, mean ± SD age at onset (AAO) 60 ± 11 years and 10% had family history of PD] and age- and population-matched 289 controls (64.8 ± 10.2 years; mean ± SD). *The PPMI cohort* was composed of 331 NHW PD cases (50% males, AAO 61 ± 11 years, 9% had family history of PD) and 134 age- and population-matched controls (60.9 ± 11.4 years). Finally, *the UN cohort* was composed of 553 Spanish PD cases (59% males, AAO 60 ± 9 years, 20% had family history of PD) and 550 healthy Spanish age-matched controls (62 ± 7 years). Only one member of each family with PD was included in the analyses. All individuals carrying pathogenic mutations in *LRRK2, p.G2019S (8 PD cases WUSLT), PARK2, p.D53X (1 PD case WUSTL), or PINK, R492X1(1 PD case WUSTL)* genes, duplications in the *SNCA* gene *(1 PD case WUSTL)* or risk-associated variants in the *TREM2, p.R47H (4 WUSTL), GBA, p.N370S (7 WUSTL)*, or *MAPT, p.A152T (4 WUSTL)* genes (Benitez and Cruchaga, 2013; Benitez et al., 2016) were excluded from this study. Principal component analyses (PCA) was conducted to infer the genetic structure of individuals using the EIGENSTRAT software (Price et al., 2006). Only subjects that clustered with PCs of NHW origin in the WUSTL or PPMI cohorts were included in this study.

### Sequencing methods

*WUSTL and UN samples:* To screen for novel variants, pooled-DNA sequencing was performed, as described previously (Jin et al., 2012; Benitez et al., 2016). Briefly, 62 PCR reactions that covered 46,319 bases of the four selected dementia genes were performed in two equimolar pools of 114 and 98 samples. After ligation, concatenated PCR products were randomly sheared by sonication and prepared for sequencing on an Illumina Genome Analyzer IIx (GAIIx) according to the manufacturer's specifications. The resulting reads were re-aligned to the human genome reference assembly build 36.1 (hg18) using SPLINTER. pCMV6-XL5 amplicon (1908 base pairs) was included in the reaction as a negative control. As positive controls, 10 different constructs (p53 gene) with synthetically engineered mutations at a relative frequency of one mutated copy per 200 normal copies was amplified and pooled with the PCR products. Six human DNA samples heterozygous for previously known mutations in *GRN* and *PSEN1*genes were also included (Jin et al., 2012; Benitez et al., 2016). SPLINTER uses the positive control to estimate sensitivity and specificity for variant calling. The wild-type: mutant ratio in the positive control is similar to the relative frequency expected for a single mutation in one pool (1 chromosome mutated in 100 samples = 1/200). SPLINTER uses the negative control (first 900 bp) to model the errors across the 36-bp Illumina reads and to create an error model from each sequencing run of the machine. Based on the error model SPLINTER calculates a *p*-value for the probability that a predicted variant is a true positive. A *p*-value at which all mutants in the positive controls were identified was defined as the cut-off value for the best sensitivity and specificity. All mutants included as part of the amplified positive control vector were found upon achieving ~30-fold coverage at mutated sites (sensitivity = 100%) and ~80 sites in the 1908 bp negative control vector were predicted to be polymorphic (specificity = ~95%). The variants with a *p*-value below this cut-off value were considered for follow-up confirmation. An average coverage of 30X-fold per haploid genome per pool is the minimum coverage necessary to get an optimal positive predictive value for the SNP-calling algorithm (Vallania et al., 2010). Supplementary Table 1 contains a summary of exon coverage per gene. On average, the coverage was 59.5x per allele per individual, which translate to a total coverage of ~13,566x or ~11,662x depending on the pool size. All evaluated variants were validated by genotyping with Sequenom iPLEX or KASPar techniques in all samples. Common variants (>5%) or synonymous variants were not followed up. *APOE* genotype was obtained by direct genotyping of rs7412 and rs429358 using Taqman technology (Cruchaga et al., 2012b). Most of rare variants were also validated by genome-wide data generated with the NeuroX custom chip in the WUSTL samples. NeuroX chip includes both the standard Illumina exome content (≈ 240,000 variants) and over 24,000 variants associated with neurologic diseases (Nalls et al., 2015). *PPMI Sample:* The VCF files containing the whole exome sequence for all PPMI samples were downloaded from the PPMI website (www.ppmi-info.org). The regions of interest for dementia-related genes were extracted from those files for further analysis. *APOE* genotype (rs7412 and rs429358) was obtained from the VCF files.

### Bioinformatics and statistical analyses

#### Annotation and variant selection

Variants were annotated using SeattleSeq Annotation (Ng et al., 2009), the Exome Variant Server (EVS), (http://evs.gs.washington.edu/EVS/ - Release ESP6500SI-V2), the ExAC data (release 0.3.1) (Lek et al., 2016) and the Ensembl Genome browser (Aken et al., 2016). Predictions of variant pathogenicity were based on the Genomic Evolutionary Rate Profiling (GERP) (Davydov et al., 2010) and the Combined Annotation Dependent Depletion (CADD) algorithm (v1.3, http://cadd.gs.washington.edu) (Kircher et al., 2014). All putative damaging variants (scores GERP ≥ 2.95 and CADD ≥ 12.37) (Amendola et al., 2015) were further cross-referenced with The AD&FTD mutation database (Cruts et al., 2012) and ClinVar (Landrum et al., 2016) to identify those previously established with pathogenicity for AD or FTD. Only variants annotated or predicted to be coding variants [missense, nonsense (stop/start gain/loss) and frameshift variants] were included in the analyses. Synonymous variants were not included in our analyses. This analysis was applied independently to all three cohorts.

#### Single variant analysis

Association analysis between PD cases and in-house controls was performed using logistic regression, assuming an additive model for allelic effects. The analysis was adjusted for age, gender, and PCs (using the first two principal components) to correct for potential population stratification using Plink1.9 (Purcell et al., 2007; Chang et al., 2015). The minor allele frequency (MAF) of each variant in PD cases was also compared with the MAF described in the Non-Finnish European subgroup (NFE) from ExAC (Lek et al., 2016). Only coding regions with high-quality (PASS filter) variants reported in ExAC were included in our analyses.

#### Gene based analysis

The gene-based association in PD cases and in-house controls was performed using SKAT-O, which utilizes the R package SKAT (Wu et al., 2011). In addition, the burden of rare protein-altering variants in PD cases from the WUSTL and PPMI cohorts were compared with the burden observed in NFE samples from ExAc by collapsing the counts of all missense, nonsense and frameshift variants in each gene with a MAF < 0.01 and then, Fisher's exact test with Yates correction was applied. ExAC cannot be regarded as a pure control dataset. However, several studies have used this resource as a proxy for reassessing the effect of rare variants in Mendelian genes in the general population in different complex diseases (Roberts et al., 2015; Walsh et al., 2017).

#### Cognitive impairment assessment and analysis

Cognitive impairment was assessed by the Folstein Mini-Mental State Examination (MMSE) and the Montreal Cognitive Assessment (MoCA) in the WUSTL and PPMI datasets respectively. For the combined analyses, z scores were derived by converting the mean raw MoCA and MMSE scores and standard deviation (SD) to the standard normal distribution with mean 0 and SD 1. Multiple regression analyses were performed in the residuals adjusting by age at diagnosis, age at dementia assessment, gender, study site and *APOE* carrier status as covariates (PROC GLM, SAS). All validated variants were included in the model independently of their clinical interpretation. Global cognitive impairment was defined by an MMSE ≤ 25 according to the recommendation the International Parkinson and Movement Disorder Society (MDS) Task Force (Dubois et al., 2007). The recommended MoCA cut-off for PD of <26 was applied (Dalrymple-Alford et al., 2010). Patients with a diagnosis of dementia [PPMI (*N* = 5) and WUSTL (*N* = 9)] were excluded from the analyses to avoid disproportionate leverage on the statistical models (Thaler et al., 2012). Nominally significant *p*-value threshold was set at 0.05. Multiple-test correction cutoff for the single-variant analysis using Bonferroni correction for 4 tests is 1.3 × 10^−2^. All statistical tests are two-sided unless otherwise stated, and were performed using Statistical Analysis System (SAS Institute Inc) or GraphPad Prism 5.0.

#### APOE analysis

The effect of *APOE* allele and genotype in PD risk was tested comparing the frequency in PD cases and controls. Comparisons were made using the *X*^2^ method. Multiple regression adjusting by age at diagnosis, age at dementia assessment and gender was performed to evaluate the effect of *APOE* ϵ4 allele on MoCA or MMSE scores (PROC GLM, SAS).

## Results

### Single variant analyses

#### WUSTL cohort

Twenty-one coding variants were found among the screened genes (Supplementary Table 2). 14.3% (3) variants were novel, 4.8% (1) were reported as known pathogenic variants, 19% (4) were classified as pathogenic nature unclear and 33% (7) were reported as non-pathogenic. The three novel variants were *PSEN1* (p.P303L), *PSEN2* (p.C358R), and *GRN* (p.A29V). Both the *PSEN1* and *PSEN2* variants were found in late-onset PD individuals whereas the *GRN* variant was found in one control (Supplementary Table 3A). The *PSEN1* p.A79V mutation (a known AD pathogenic mutation) was found in three PD cases: An early-onset PD case (44 years old at onset) and two late-onset PD cases (75 and 64 years old at onset, respectively). None of the three carriers reported PD or AD family history. Neurological evaluation of these three patients at an age of 46, 86, and 82 years revealed no evidence of dementia after 2, 11, and 18 years of disease onset, respectively. The carrier of the “probable pathogenic” PSEN2, p.S130L variant exhibited an AAO of 45 years and rapid progression of cognitive impairment (Supplementary Table 3B).

#### PPMI cohort

Eighteen coding variants were found in 5% of the cases and 8.9% of the controls (Supplementary Table 4). 22% (4) of the variants were novel, 28% (5) were classified as pathogenic nature unclear and 28% (5) were reported as non-pathogenic; 22% (4) were reported in public databases but with an unknown clinical significance. The four variants of unknown significance included *APP* (p.R499C), *APP* (p.R397T), *APP* (p.Q138R), and *GRN* (p.C260R). Both *APP* p.R499C and p.R397T were found in late-onset PD (LOPD; AAO >50 years) patients whereas *APP* (p.Q138R) and *GRN* (p.C260R) were found in controls (Supplementary Table 5). Five variants in the *PSEN2* gene were considered of pathogenic nature unclear for AD: p.R62H, p.R71W, p.S130L, p.K161R, and p.V393M. Two LOPD patients carried *PSEN2* p.S130L variant and were cognitively normal at the last clinical assessment (Supplementary Table 5). Interestingly, the *PSEN2* p.R71W variant was present in five controls and two PD patients achieving nominal significance (*p* = 0.01; *OR* = 0.16; 95% *CI* = 0.02–0.98).

#### UN cohort

Ten coding variants were found in 4.9% of cases from the Spanish PD cohort. No novel variants were found (Supplementary Table 6). Nine variants were present in the AD/FTD mutation database. 50% (5) of them were considered non-pathogenic, 30% (3) of them were of pathogenic nature unclear, and 10% (1) of them (*PSEN2* p.V148I) was considered to be pathogenic (Cruts et al., 2012). The *PSEN2* p.V148I carrier is an early onset PD case (25 years at onset) with a tremor-dominant parkinsonism and positive family history of PD but dementia-free at last assessment after 12 years of PD. Seven (1.3%) carriers of the *PSEN2* p.S130L variant were found in Spanish PD patients. Three had positive PD family history, three had EOPD, three had psychiatric co-morbidities and two with dementia PD (Supplementary Table 7).

### Gene-based analysis

The gene-based analysis is a powerful tool to uncover genetic association. In previous studies, we used gene-based analysis of the *GBA* gene in PD and *TREM2* in AD to identify additional variants associated with risk (Jin et al., 2015; Benitez et al., 2016). SKAT-O analysis revealed that none of the dementia-related genes achieved statistical significance in the WUSTL series *[APP* (*p* = 0.89), *GRN* (*p* = 0.63), *PSEN1* (*p* = 0.13), and *PSEN2* (*p* = 0.5)] or PPMI [*APP* (*p* = 0.15), *GRN* (*p* = 0.6), *PSEN1* (*p* = 0.9) and *PSEN2* (*p* = 0.09)] compared with in-house controls (Supplementary Tables 2, 4). However, joint burden analysis revealed a significant enrichment of rare variants in the *GRN* (6.6 × 10^−03^; *OR* = 1.9; 95% *CI* = 1.2–3.0) and *PSEN1* (*p* = 9.2 × 10^−41^; *OR* = 54.2; 95% *CI* = 18.8–156.1) genes in PD cases compared with the ExAC NFE cohort (Table 1). The association of *PSEN1* and PD was maintained (*p* = 4.9 × 10^−66^) after excluding the *PSEN1* p.A79V mutation from the analysis.

**Table 1:** Enrichment of rare variants in the *PSEN1* and *GRN* genes in PD patients.

**Gene**	cMAF^*^ PD Cases	**cMAF ExAC^φ^**	***p*-value^#^**	**OR (95% CI)^#^**
*APP*	0.0004	0.0004	ns^§^	-
*GRN*	0.0010	0.0005	6.6 × 10^−03^	1.9 (1.2–3.0)
*PSEN1*	0.0010	0.00002	9.1 × 10^−41^	54.2 (18.8–156.1)
*PSEN2*	0.0010	0.0010	ns	-

* *cMAF = cumulative minor allele frequency of all non-synonymous variants in each gene*.

φ *Non-Finnish European ExAC individuals*.

# *WUSTL and PPMI PD cases vs ExAC non-Finish Europeans controls*.

§ *Not statistically significant*.

### APOE association with status and cognitive test performance

*APOE* has previously been associated with cognitive impairment in PD patients (Parsian et al., 2002; Morley et al., 2012; Tsuang et al., 2013) but APOE effect on PD risk is still controversial (Federoff et al., 2012). Here, no association was found between different *APOE* alleles (ε4 or ε2) and PD case-control status (Supplementary Tables 8A,B). The *APOE* ε4 allele was not associated with lower MoCA scores among PD patients in the PPMI cohort (*p* = 0.56). However, consistent with a previous report, the presence of the *APOE* ε4 allele was associated with a lower MMSE score (*p* = 9.0 × 10^−3^) in WUSTL PD patients (Parsian et al., 2002).

### Effect of variants in dementia-causing genes on cognitive tests performance

**Figure 1:**
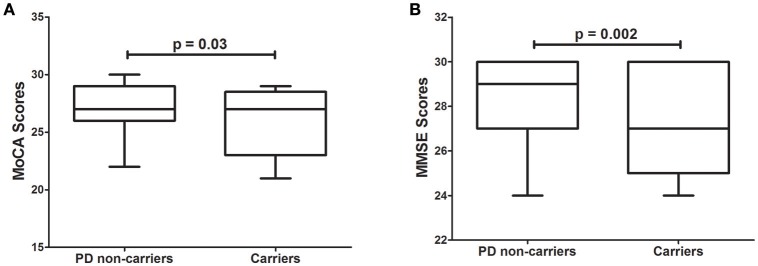
Effect of variants in *APP, GRN, PSEN1*, and *PSEN2* genes on cognitive test scores in PD patients. **(A)**, Tukey's boxplot of MoCA scores in PD non-carriers vs. PD carriers of variants in the dementia-causing genes in the PPMI cohort. **(B)**, Tukey's boxplot of the MMSE scores in non-carriers and carriers of variants in the dementia-causing genes in the PD cases of the WUSTL cohort. Student's *t*-test was used and *p*-values reported are based on two-tailed comparisons.

Several rare variants in the *GBA* gene have been associated with lower MMSE scores in PD patients (Liu et al., 2016). We hypothesized that variants in the dementia-causing genes would affect performance on cognitive tests. Interestingly, 4.9% of all PD patients in the PPMI cohort carrying rare variants in dementia-causing genes exhibit significantly (*p* = 3.0 × 10^−2^) lower MoCA scores than non-carriers PD patients (Figure 1A). Similarly, PD patients carrying rare variants in dementia-causing genes (7.9% of all patients) exhibit significantly (*p* = 2.0 × 10^−3^) lower MMSE scores than non-carriers in the WUSTL cohort (Figure 1B). Cognitive impairment was assessed with a different cognitive test in each cohort included in this study (MMSE for WUSTL and MoCA for PPMI). Therefore, we perform a combined analysis with age at PD diagnosis, age at which the dementia test was performed, *APOE* status, and cohort included as covariates. PD patients carrying rare variants in dementia-causing genes exhibited lower scores on cognitive tests than non-carrier PD patients (*p* = 2.0 × 10^−4^), independent of age at PD diagnosis, age at evaluation, *APOE* status or cohort (Table 2).

**Table 2:** Effect of rare variants in dementia-causing genes on cognitive test scores in PD patients.

**Outcome**	**Independent variable**	**Estimate**	**Standard error**	***t*-value**	***p*-value**
Cognitive test score^φ^	Age at PD Dx^§^	0.006	0.009	0.64	0.5225
	Age at assessment	−0.015	0.009	−1.64	0.1018
	APOE^*^	−0.039	0.041	−0.96	0.3385
	Carrier status^#^	−0.684	0.180	−3.80	0.0002
	Recruitment sites^φ^	0.223	0.142	1.58	0.1153

§ *Dx = diagnostic*.

φ *Combined analysis MoCA and MMSE*.

* *APOE genotype 22 = 0, 23 = 1, 33 = 2, 24 = 3, 34 = 4, and 44 = 5*.

# *0 = non carrier, 1 = carrier*.

φ *0 = PPMI, 1 = WUSTL*.

## Discussion

The genetic architecture of dementia in PD has not yet been fully established. Genetic variants in *APOE* and *MAPT* have time-dependent effects on cognition, which vary with disease stage: *MAPT* appears to have its greatest impact on cognitive decline in early PD, whereas *APOE* may have a more pronounced effect late in the course of the disease (Collins and Williams-Gray, 2016). A recent GWAS on an extensive neuropsychological battery in PD patients failed to replicate prior associations with *APOE, MAPT, catechol-O-methyltransferase (COMT)*, or *SNCA* (Mata et al., 2017). In cross-sectional studies, the prevalence of dementia in *GBA*-PD cases is about 50%, compared to 24–31% in idiopathic PD cases (Setó-Salvia et al., 2012). Longitudinal studies have confirmed a faster progression to dementia in PD cases carrying *GBA* mutations compared to idiopathic PD in a population-representative cohort followed for ~10 years from diagnosis (Winder-Rhodes et al., 2013). However, *GBA* variants explained only up to 1.4% of PD patients with cognitive decline (Liu et al., 2016). Our results show that 6.7% of PD patients carry rare coding variants in dementia-causing genes and exhibit lower scores on cognitive tests in two independent cohorts compared to non-carriers. The risk of developing dementia varies according to the duration of PD and age at onset (Rocca et al., 2007). However, the results of a covariate-adjusted model confirm that the association between rare variants in dementia-causing genes and lower scores in cognitive tests appears to be independent of age, cohort, disease duration, or *APOE* status.

The *APOE* ε4 allele has been associated with a higher prevalence of dementia in PD (Morley et al., 2012; Tsuang et al., 2013). The small number of PD cases with dementia in each study, the significant heterogeneity of odds ratios between studies, and evidence of publication bias limits the confidence of the *APOE* and dementia in PD association (Huang et al., 2006). In this study, the *APOE* ε4 allele was not associated with risk of developing PD in none of the cohorts (Supplementary Tables 8A,B). However, in the WUSTL cohort, *APOE* ε4 carriers exhibited lower scores on cognitive tests, but the PPMI cohort did not replicate these findings. Therefore, further studies are needed to clarify the role of *APOE* in PD cognitive impairment (Parsian et al., 2002).

The wide variation reported in the prevalence of cognitive impairment (CI) in PD across studies may be due to the cognitive tests employed (Goldman and Litvan, 2011; Burdick et al., 2014). The MMSE and MoCA are the most commonly used tests to assess CI in PD. However, controversy remains regarding the sensitivity of MMSE in assessing cognition in PD patients (Burdick et al., 2014). Even though the MoCA is more sensitive for detecting cognitive changes in PD patients (Hoops et al., 2009), the MMSE was the cognitive test that showed the effects of *GBA* and *LRRK2* mutations on CI in PD patients (Srivatsal et al., 2015; Liu et al., 2016) and is the cognitive test recommended by the International Parkinson and Movement Disorder Society Task Force (Dubois et al., 2007). Nevertheless, here we show that rare variants in dementia-related genes affect both MMSE and MoCA scores in two heterogeneous PD populations (*p* = 2.0 × 10^−4^; Table 2).

In addition, 0.3% of all PD patients screened in this study carry known AD pathogenic variants in *PSEN1* and *PSEN2* genes (Supplementary Tables 2, 6). A frequency of 0.3% is comparable to the percentage of PD patients carrying the most common pathogenic mutation (*LRRK2* p.G2019S) known for sporadic PD (0.4–1%) (Healy et al., 2008; Correia Guedes et al., 2010). None of the PD patients carrying pathogenic mutations in *PSEN1* and *PSEN2* exhibited signs of dementia at their last clinical assessment. Unfortunately, CSF biomarkers or Pittsburgh compound B (PiB) imaging were not available from these carriers to help determine their preclinical status. The *PSEN1* p.A79V mutation is associated with a broad range of AAO in AD and DLB patients (Cruchaga et al., 2012a; Meeus et al., 2012b). *PSEN1* p.A79V is found in non-demented carriers at an AAO as late as 78 years (Kauwe et al., 2007) and, in some multigenerational AD families, it does not segregate perfectly with disease status (Cruchaga et al., 2012a). However, the *PSEN1* p.A79V mutation increases the Aβ42 level and Aβ42/Aβ40 ratio *in vitro* (Kumar-Singh et al., 2006; Kauwe et al., 2007) but unlike other *PSEN1* mutations that cause massive Aβ42 deposition, the average Aβ42 brain deposition in sporadic AD patients exceeded those observed in the brains of p.A79V carriers (Kumar-Singh et al., 2006). In addition, non-demented p.A79V carriers also exhibit changes in CSF Aβ42 levels and the Aβ42/40 ratio with no evidence of β-amyloid deposition using PiB imaging (Kauwe et al., 2007). Thus, *PSEN1* p.A79V may be acting through a mechanism other than increasing Aβ deposition. Additional mutations in the *PSEN1* gene including p.G217D, p.V272A, and p.L420R have been associated with dementia and parkinsonism (Takao et al., 2002; Jimenez-Escrig et al., 2004; Puschmann et al., 2009; Niwa et al., 2013). Recently, it was reported that a *de novo PSEN1* mutation is responsible for an early-onset parkinsonism with cognitive impairment (Carecchio et al., 2017). In addition to the clinical overlap between dementia and parkinsonism in carriers of *PSEN1* mutations, there is extensive Lewy body pathology in early-onset AD carriers of the *PSEN1* p.S170F (Snider et al., 2005) and *PSEN1* p.A431V mutations (Qiao et al., 2017) suggesting an interaction between *PSEN1* dysfunction and α-synuclein aggregation.

We also found the *PSEN2* p.V148I mutation in a Spanish PD patient but not in 550 age-matched Spanish controls. The *PSEN2* p.V148I mutation was originally reported in a Spanish patient with late-onset AD (AAO = 71 years) (Lao et al., 1998) but, its pathogenicity has been questioned based on the absence of effect on either Aβ42 levels, Aβ40 levels, or the Aβ42/40 ratio *in vitro* (Walker et al., 2005). However, some variants that show no effect on Aβ42 levels or the Aβ42/Aβ40 ratio (Walker et al., 2005) affect calcium signaling in cultured skin fibroblasts from mutation carriers (Li et al., 2006). Increasing evidence suggests a role of the *PSEN2* p.S130L variant in PD and dementia. Here, we found a total of nine PD patients and one control carrying the “probably pathogenic” *PSEN2* p.S130L variant. The *PSEN2* p.S130L variant has been reported in patients with late-onset AD and mild bradykinesia (Tomaino et al., 2007; Lohmann et al., 2012). Two siblings of a *PSEN2* p.S130L carrier AD patient were diagnosed with PD (Tomaino et al., 2007). Recently, *PSEN2* p.S130L was reported in an individual with idiopathic PD with dementia (AAO = 73 years) (Schulte et al., 2015). Another mutation in *PSEN2* (p.V191E) also was found in one late-onset PD patient (AAO = 75 years) with cognitive decline (Meeus et al., 2012b). Interestingly, a Swedish PD family who carry a *de novo* α-synuclein p.A53T mutation also carried the *PSEN2* p.R163H variant (Puschmann et al., 2009). Carriers of both mutations develop early-onset dementia (Puschmann et al., 2009). All these findings support our data that pathogenic variants in the presenilin genes are present in a small proportion of sporadic PD patients and contribute to α-synuclein aggregation.

We found an enrichment of rare variants in the *GRN* gene in PD patients compared to the general population (ExAC NFE). The frequencies of the p.R433W and p.R478H variants were higher in the WUSTL cohort than in the NFE ExAC cohort. Interestingly, the variant p.R433W was reported in neuropathologically confirmed LBD cases (Meeus et al., 2012b). Mutations in the *GRN* gene occur in LBD patients (Meeus et al., 2012b). A heterozygous deletion removing exons 1 to 11 of the *GRN* gene was reported in an 83 year-old PD patient (Rovelet-Lecrux et al., 2008) and, the IVS0 + 5G>C mutation was reported in a 56 year-old PD patient (Brouwers et al., 2007). In addition, parkinsonism occurs in some FTD patients and is more common in those patients (up to 41%) with *GRN* haploinsufficiency (Josephs et al., 2007). Interestingly, *GRN* overexpression in the substantia nigra protected nigrostriatal neurons in a mouse model of PD (Van Kampen et al., 2014). *GRN* seems to play an important role in multiple neurodegenerative diseases including PD, likely due to its function as a neurotrophic factor and its recently uncovered lysosome function (Tanaka et al., 2013).

Low frequency and rare mutations in the *GBA* gene, which encodes the lysosomal enzyme β-glucocerebrosidase-1, consistently relate to CI in PD and to Lewy body dementia (Nalls et al., 2013). α-synuclein is mainly degraded by lysosomes (Cuervo et al., 2004) and lysosomal dysfunction may contribute to *de novo* aggregation of α-synuclein and impaired autophagic degradation of cytosolic aggregates (Bourdenx et al., 2014). LBs and Lewy neurites may seed around impaired lysosomes and grow in size by continuous deposition of lysosomal-derived un-degraded material as the disease progresses (Dehay et al., 2013). Thus, considering that presenilin and granulin are lysosomal proteins (Sannerud et al., 2016; Kao et al., 2017), it is logical to suggest that variants in the presenilin or granulin genes may exacerbate the cognitive impairments in PD by affecting lysosomal function and facilitating cell-to-cell transfer of proteopathic seeds in the progression of synucleinopathies.

The relatively small size of this study limits the statistical power, which could be the reason we failed to find significant associations between the PD cases and the in-house controls. However, the inclusion of the large NFE ExAC cohort with similar genetic background minimized this limitation. The data used in this study were not generated using a single sequencing method. However, none of the approaches used were expected to have 100% sensitivity for variant detection. Although these technical limitations could have marginal effects on estimates of rare variant frequency and odds ratio values, we do not expect them to alter the key conclusions of this study. Further studies are needed to confirm the role of variants in dementia genes in the cognitive impairment found in PD patients.

## Conclusion

Our study shows that rare variants in several dementia-related genes are enriched in PD patients compared with normal controls. The PD patients with these variants exhibited lower cognitive performance than PD patients without these variants. Moreover, known dementia-causing mutations are found in PD patients.

## Author contributions

BB and CC conceived and designed the study. JP, AD, UD, SO-C, and PP acquired and analyzed the clinical data. LI, BB, MF, JB, and MD-F acquired the genetic data. LI, BB, and CC performed the statistical analysis and interpreted the genetic data. LI and BB wrote the draft of the manuscript and JP, AD, PP, and CC provided critical comments on the draft of the manuscript. All authors read and approved the final version of the manuscript.

### Conflict of interest statement

The authors declare that the research was conducted in the absence of any commercial or financial relationships that could be construed as a potential conflict of interest.

## References

[B1] Aarsland D. Kurz M. W. (2010). The epidemiology of dementia associated with Parkinson's disease. Brain Pathol. 20, 633–639. 10.1111/j.1750-3639.2009.00369.x 20522088 PMC8094858

[B2] Aken B. L. Ayling S. Barrell D. Clarke L. Curwen V. Fairley S.. (2016). The Ensembl gene annotation system. Database 2016:baw093. 10.1093/database/baw093 27337980 PMC4919035

[B3] Amendola L. M. Dorschner M. O. Robertson P. D. Salama J. S. Hart R. Shirts B. H.. (2015). Actionable exomic incidental findings in 6503 participants: challenges of variant classification. Genome Res. 25, 305–315. 10.1101/gr.183483.114 25637381 PMC4352885

[B4] Baizabal-Carvallo J. F. Jankovic J. (2016). Parkinsonism, movement disorders and genetics in frontotemporal dementia. Nat. Rev. Neurol. 12, 175–185. 10.1038/nrneurol.2016.14 26891767

[B5] Benitez B. A. Cruchaga C. (2013). TREM2 and neurodegenerative disease. N. Engl. J. Med. 369, 1567–1578 10.1056/NEJMc1306509#SA4 24131187 PMC4380008

[B6] Benitez B. A. Davis A. A. Jin S. C. Ibanez L. Ortega-Cubero S. Pastor P.. (2016). Resequencing analysis of five Mendelian genes and the top genes from genome-wide association studies in Parkinson's disease. Mol. Neurodegener. 11:29. 10.1186/s13024-016-0097-0 27094865 PMC4837564

[B7] Bourdenx M. Bezard E. Dehay B. (2014). Lysosomes and Î±-synuclein form a dangerous duet leading to neuronal cell death. Front. Neuroanat. 8:83. 10.3389/fnana.2014.00083 25177278 PMC4132369

[B8] Brouwers N. Nuytemans K. van der Zee J. Gijselinck I. Engelborghs S. Theuns J.. (2007). Alzheimer and Parkinson diagnoses in progranulin null mutation carriers in an extended founder family. Arch. Neurol. 64, 1436–1446. 10.1001/archneur.64.10.1436 17923627

[B9] Burdick D. J. Cholerton B. Watson G. S. Siderowf A. Trojanowski J. Q. Weintraub D.. (2014). People with Parkinson's disease and normal MMSE score have a broad range of cognitive performance. Mov. Disord. 29, 1258–1264. 10.1002/mds.25924 25073717 PMC4162839

[B10] Carecchio M. Picillo M. Valletta L. Elia A. E. Haack T. B. Cozzolino A.. (2017). Rare causes of early-onset dystonia-parkinsonism with cognitive impairment: a *de novo* PSEN-1 mutation. Neurogenetics 18, 175–178. 10.1007/s10048-017-0518-4 28664294

[B11] Chang C. C. Chow C. C. Tellier L. C. Vattikuti S. Purcell S. M. Lee J. J. (2015). Second-generation PLINK: rising to the challenge of larger and richer datasets. Gigascience 4:7. 10.1186/s13742-015-0047-8 25722852 PMC4342193

[B12] Chang D. Nalls M. A. Hallgrímsdóttir I. B. Hunkapiller J. van der Brug M. Cai F.. (2017). A Meta-analysis of genome-wide association studies identifies 17 new Parkinson's disease risk loci. Nat. Genet. 49, 1511–1516. 10.1038/ng.3955 28892059 PMC5812477

[B13] Collins L. M. Williams-Gray C. H. (2016). The genetic basis of cognitive impairment and dementia in parkinson's disease. Front. Psychiatry 7:89. 10.3389/fpsyt.2016.00089 27242557 PMC4873499

[B14] Compta Y. Parkkinen L. O'Sullivan S. S. Vandrovcova J. Holton J. L. Collins C.. (2011). Lewy- and Alzheimer-type pathologies in Parkinson's disease dementia: Which is more important? Brain 134, 1493–1505. 10.1093/brain/awr031 21596773 PMC4194668

[B15] Correia Guedes L. Ferreira J. J. Rosa M. M. Coelho M. Bonifati V. Sampaio C. (2010). Worldwide frequency of G2019S LRRK2 mutation in Parkinson's disease: A systematic review. Park. Relat. Disord. 16, 237–242. 10.1016/j.parkreldis.2009.11.004 19945904

[B16] Cruchaga C. Chakraverty S. Mayo K. Vallania F. L. Mitra R. D. Faber K.. (2012a). Rare variants in APP, PSEN1 and PSEN2 increase risk for AD in late-onset Alzheimer's disease families. PLoS ONE 7:e31039. 10.1371/journal.pone.0031039 22312439 PMC3270040

[B17] Cruchaga C. Kauwe J. S. Nowotny P. Bales K. Pickering E. H. Mayo K.. (2012b). Cerebrospinal fluid APOE levels: An endophenotype for genetic studies for Alzheimer's disease. Hum. Mol. Genet. 21, 4558–4571. 10.1093/hmg/dds296 22821396 PMC3459471

[B18] Cruts M. Theuns J. Van Broeckhoven C. (2012). Locus-specific mutation databases for neurodegenerative brain diseases. Hum. Mutat. 33, 1340–1344. 10.1002/humu.22117 22581678 PMC3465795

[B19] Cuervo A. M. Stefanis L. Fredenburg R. Lansbury P. T. Sulzer D. (2004). Impaired degradation of mutant alpha-synuclein by chaperone-mediated autophagy. Science 305, 1292–1295. 10.1126/science.1101738 15333840

[B20] Dalrymple-Alford J. C. MacAskill M. R. Nakas C. T. Livingston L. Graham C. Crucian G. P.. (2010). The MoCA: Well-suited screen for cognitive impairment in Parkinson disease. Neurology 75, 1717–1725. 10.1212/WNL.0b013e3181fc29c9 21060094

[B21] Davis A. A. Andruska K. M. Benitez B. A. Racette B. A. Perlmutter J. S. Cruchaga C. (2015). Variants in GBA, SNCA, and MAPT influence Parkinson disease risk, age at onset, and progression. Neurobiol. Aging. 37:209.e1–209.e7. 10.1016/j.neurobiolaging.2015.09.014 26601739 PMC4688052

[B22] Davydov E. V. Goode D. L. Sirota M. Cooper G. M. Sidow A. Batzoglou S. (2010). Identifying a high fraction of the human genome to be under selective constraint using GERP++. PLoS Comput. Biol. 6:e1001025. 10.1371/journal.pcbi.1001025 21152010 PMC2996323

[B23] Dehay B. Martinez-Vicente M. Caldwell G. A. Caldwell K. A. Yue Z. Cookson M. R.. (2013). Lysosomal impairment in Parkinson's disease. Mov. Disord. 28, 725–732. 10.1002/mds.25462 23580333 PMC5131721

[B24] Dubois B. Burn D. Goetz C. Aarsland D. Brown R. G. Broe G. A.. (2007). Diagnostic procedures for Parkinson's disease dementia: recommendations from the movement disorder society task force. Mov. Disord. 22, 2314–2324. 10.1002/mds.21844 18098298

[B25] Emre M. Aarsland D. Brown R. Burn D. J. Duyckaerts C. Mizuno Y.. (2007). Clinical diagnostic criteria for dementia associated with Parkinson's disease. Mov. Disord. 22, 1689–1707. 17542011 10.1002/mds.21507

[B26] Federoff M. Jimenez-Rolando B. Nalls M. A. Singleton A. B. (2012). A large study reveals no association between APOE and Parkinson's disease. Neurobiol. Dis. 46, 389–392. 10.1016/j.nbd.2012.02.002 22349451 PMC3323723

[B27] Fernández M. V. Kim J. H. Budde J. P. Black K. Medvedeva A. Saef B.. (2017). Analysis of neurodegenerative Mendelian genes in clinically diagnosed Alzheimer Disease. PLoS Genet. 13:e1007045. 10.1371/journal.pgen.1007045 29091718 PMC5683650

[B28] Ferrari R. Wang Y. Vandrovcova J. Guelfi S. Witeolar A. Karch C. M.. (2017). Genetic architecture of sporadic frontotemporal dementia and overlap with Alzheimer's and Parkinson's diseases. J. Neurol. Neurosurg. Psychiatry 88, 152–164. 10.1136/jnnp-2016-314411 27899424 PMC5237405

[B29] Foltynie T. Brayne C. E. G. Robbins T. W. Barker R. A. (2004). The cognitive ability of an incident cohort of Parkinson's patients in the UK. The CamPaIGN study. Brain 127, 550–560. 10.1093/brain/awh067 14691062

[B30] Goldman J. G. Litvan I. (2011). Mild cognitive impairment in Parkinson's disease. Minerva Med. 102, 441–459. 22193376 PMC3370887

[B31] Guerreiro R. Escott-Price V. Darwent L. Parkkinen L. Ansorge O. Hernandez D. G.. (2015). Genome-wide analysis of genetic correlation in dementia with Lewy bodies, Parkinson's and Alzheimer's diseases. Neurobiol. Aging. 38, 214.e7–214.e10. 10.1016/j.neurobiolaging.2015.10.028 26643944 PMC4759606

[B32] Harms M. B. Neumann D. Benitez B. A. Cooper B. Carrell D. Racette B. A. (2013). Parkinson disease is not associated with C9ORF72 repeat expansions. Neurobiol. Aging 34:1519.e1-2 10.1016/j.neurobiolaging.2012.10.001 PMC3566343 23116878

[B33] Healy D. G. Falchi M. O'Sullivan S. S. Bonifati V. Durr A. Bressman S.. (2008). Phenotype, genotype, and worldwide genetic penetrance of LRRK2-associated Parkinson's disease: a case-control study. Lancet Neurol. 7, 583–590. 10.1016/S1474-4422(08)70117-0 18539534 PMC2832754

[B34] Hoops S. Nazem S. Siderowf A. D. Duda J. E. Xie S. X. Stern M. B.. (2009). Validity of the MoCA and MMSE in the detection of MCI and dementia in Parkinson disease. Neurology 73, 1738–1745. 10.1212/WNL.0b013e3181c34b47 19933974 PMC2788810

[B35] Huang X. Chen P. Kaufer D. I. Tröster A. I. Poole C. (2006). Apolipoprotein E and dementia in Parkinson disease: a meta-analysis. Arch. Neurol. 63, 189–193. 10.1001/archneur.63.2.189 16476806

[B36] Hughes A. J. Daniel S. E. Kilford L. Lees A. J. (1992). Accuracy of clinical diagnosis of idiopathic Parkinson's disease: a clinico-pathological study of 100 cases. J. Neurol. Neurosurg. Psychiatry 55, 181–184. 1564476 10.1136/jnnp.55.3.181 PMC1014720

[B37] Irwin D. J. Lee V. M.-Y. Trojanowski J. Q. (2013). Parkinson's disease dementia: convergence of α-synuclein, tau and amyloid-β pathologies. Nat. Rev. Neurosci. 14, 626–636. 10.1038/nrn3549 23900411 PMC4017235

[B38] Jimenez-Escrig A. Rabano A. Guerrero C. Simon J. Barquero M. S. Güell I.. (2004). New V272A presenilin 1 mutation with very early onset subcortical dementia and parkinsonism. Eur. J. Neurol. 11, 663–669. 10.1111/j.1468-1331.2004.00865.x 15469450

[B39] Jin S. C. Carrasquillo M. M. Benitez B. A. Skorupa T. Carrell D. Patel D.. (2015). TREM2 is associated with increased risk for Alzheimer's disease in African Americans. Mol. Neurodegener. 10, 19. 10.1186/s13024-015-0016-9 25886450 PMC4426167

[B40] Jin S. C. Pastor P. Cooper B. Cervantes S. Benitez B. A. Razquin C.. (2012). Pooled-DNA sequencing identifies novel causative variants in PSEN1, GRN and MAPT in a clinical early-onset and familial Alzheimer's disease Ibero-American cohort. Alzheimer Res. Ther. 4:34. 10.1186/alzrt137 22906081 PMC3506948

[B41] Josephs K. A. Ahmed Z. Katsuse O. Parisi J. F. Boeve B. F. Knopman D. S.. (2007). Neuropathologic features of frontotemporal lobar degeneration with ubiquitin-positive inclusions with progranulin gene (PGRN) mutations. J. Neuropathol. Exp. Neurol. 66, 142–151. 10.1097/nen.0b013e31803020cf 17278999

[B42] Kao A. W. McKay A. Singh P. P. Brunet A. Huang E. J. (2017). Progranulin, lysosomal regulation and neurodegenerative disease. Nat. Rev. Neurosci. 18, 325–333. 10.1038/nrn.2017.36 28435163 PMC6040832

[B43] Kauwe J. S. K. Jacquart S. Chakraverty S. Wang J. Mayo K. Fagan A. M.. (2007). Extreme cerebrospinal fluid amyloid β levels identify family with late-onset Alzheimer's disease presenilin 1 mutation. Ann. Neurol. 61, 446–453. 10.1002/ana.21099 17366635

[B44] Kircher M. Witten D. M. Jain P. O'Roak B. J. Cooper G. M. Shendure J. (2014). A general framework for estimating the relative pathogenicity of human genetic variants. Nat. Genet. 46, 310–315. 10.1038/ng.2892 24487276 PMC3992975

[B45] Kotzbauer P. T. Cairns N. J. Campbell M. C. Willis A. W. Racette B. A. Tabbal S. D.. (2012). Pathologic accumulation of α-synuclein and Aβ in Parkinson disease patients with dementia. Arch. Neurol. 69, 1326–1331. 10.1001/archneurol.2012.1608 22825369 PMC3616136

[B46] Kumar-Singh S. Theuns J. Van Broeck B. Pirici D. Vennekens K. Corsmit E.. (2006). Mean age-of-onset of familial alzheimer disease caused by presenilin mutations correlates with both increased Abeta42 and decreased Abeta40. Hum Mutat 27, 686–695. 10.1002/humu.20336 16752394

[B47] Landrum M. J. Lee J. M. Benson M. Brown G. Chao C. Chitipiralla S.. (2016). ClinVar: public archive of interpretations of clinically relevant variants. Nucleic Acids Res. 44, D862–D868. 10.1093/nar/gkv1222 26582918 PMC4702865

[B48] Lao J. I. Beyer K. Fernández-Novoa L. Cacabelos R. (1998). A novel mutation in the predicted TM2 domain of the presenilin 2 gene in a Spanish patient with late-onset Alzheimer's disease. Neurogenetics 1, 293–296. 10732806 10.1007/s100480050044

[B49] Lek M. Karczewski K. J. Minikel E. V. Samocha K. E. Banks E. Fennell T.. (2016). Analysis of protein-coding genetic variation in 60,706 humans. Nature 536, 285–291. 10.1038/nature19057 27535533 PMC5018207

[B50] Li D. Parks S. B. Kushner J. D. Nauman D. Burgess D. Ludwigsen S.. (2006). Mutations of presenilin genes in dilated cardiomyopathy and heart failure. Am. J. Hum. Genet. 79, 1030–1039. 10.1086/509900 17186461 PMC1698711

[B51] Liu G. Boot B. Locascio J. J. Jansen I. E. Winder-Rhodes S. Eberly S.. (2016). Specifically neuropathic Gaucher's mutations accelerate cognitive decline in Parkinson's. Ann. Neurol. 80, 674–685. 10.1002/ana.24781 27717005 PMC5244667

[B52] Lohmann E. Guerreiro R. J. Erginel-Unaltuna N. Gurunlian N. Bilgic B. Gurvit H.. (2012). Identification of PSEN1 and PSEN2 gene mutations and variants in Turkish dementia patients. Neurobiol. Aging 33:1850.e17–1850.e27. 10.1016/j.neurobiolaging.2012.02.020 22503161 PMC4669567

[B53] Mata I. F. Johnson C. O. Leverenz J. B. Weintraub D. Trojanowski J. Q. Van Deerlin V. M.. (2017). Large-scale exploratory genetic analysis of cognitive impairment in Parkinson's disease. Neurobiol. Aging 56, 211.e1–211.e7. 10.1016/j.neurobiolaging.2017.04.009 28526295 PMC5536182

[B54] Meeus B. Theuns J. Van Broeckhoven C. (2012a). The genetics of dementia with Lewy bodies: what are we missing? Arch. Neurol. 69, 1113–1118. 10.1001/archneurol.2011.3678 22635379

[B55] Meeus B. Verstraeten A. Crosiers D. Engelborghs S. Van den Broeck M. Mattheijssens M.. (2012b). DLB and PDD: a role for mutations in dementia and Parkinson disease genes? Neurobiol. Aging 33:629.e5–629.e18. 10.1016/j.neurobiolaging.2011.10.014 22118943

[B56] Morley J. F. Xie S. X. Hurtig H. I. Stern M. B. Colcher A. Horn S.. (2012). Genetic influences on cognitive decline in Parkinson's disease. Mov. Disord. 27, 512–518. 10.1002/mds.24946 22344634 PMC3323737

[B57] Nalls M. A. Bras J. Hernandez D. G. Keller M. F. Majounie E. Renton A. E.. (2015). NeuroX, a fast and efficient genotyping platform for investigation of neurodegenerative diseases. Neurobiol. Aging 36, 1605.e7–1605.e12. 10.1016/j.neurobiolaging.2014.07.028 25444595 PMC4317375

[B58] Nalls M. A. Duran R. Lopez G. Kurzawa-Akanbi M. McKeith I. G. Chinnery P. F.. (2013). A multicenter study of glucocerebrosidase mutations in dementia with lewy bodies. JAMA Neurol. 70, 727–735. 10.1001/jamaneurol.2013.1925 23588557 PMC3841974

[B59] Ng S. B. Turner E. H. Robertson P. D. Flygare S. D. Bigham A. W. Lee C.. (2009). Targeted capture and massively parallel sequencing of 12 human exomes. Nature 461, 272–276. 10.1038/nature08250 19684571 PMC2844771

[B60] Niwa A. Matsuo K. Shindo A. Yata K. Shiraishi T. Tomimoto H. (2013). Clinical and neuropathological findings in a patient with familial Alzheimer disease showing a mutation in the PSEN1 gene. Neuropathology 33, 199–203. 10.1111/j.1440-1789.2012.01340.x 22882713

[B61] Parsian A. Racette B. Goldsmith L. J. Perlmutter J. S. (2002). Parkinson's disease and apolipoprotein E: possible association with dementia but not age at onset. Genomics 79, 458–461. 10.1006/geno.2002.6707 11863377

[B62] Pastor P. Ezquerra M. Muñoz E. Martí M. J. Blesa R. Tolosa E.. (2000). Significant association between the tau gene A0/A0 genotype and Parkinson's disease. Ann. Neurol. 47, 242–245. 10.1002/1531-8249(200002)47:2<242::AID-ANA16>3.0.CO;2-L 10665497

[B63] Petrucci S. Consoli F. Valente E. M. (2014). Parkinson Disease genetics: a “Continuum” from mendelian to multifactorial inheritance. Curr. Mol. Med. 14, 1079–1088. 10.2174/1566524014666141010155509 25323865

[B64] Price A. Patterson N. J. Plenge R. M. Weinblatt M. E. Shadick N. Reich D. (2006). Principal components analysis corrects for stratification in genome-wide association studies. Nat. Genet. 38, 904–909. 10.1038/ng1847 16862161

[B65] Purcell S. Neale B. Todd-Brown K. Thomas L. Ferreira M. A. R. Bender D.. (2007). PLINK: A tool set for whole-genome association and population-based linkage analyses. Am. J. Hum. Genet. 81, 559–575. 10.1086/519795 17701901 PMC1950838

[B66] Puschmann A. Ross O. A. Vilariño-Güell C. Lincoln S. J. Kachergus J. M. Cobb S. A.. (2009). A Swedish family with *de novo* α-synuclein A53T mutation: Evidence for early cortical dysfunction. Park. Relat. Disord. 15, 627–632. 10.1016/j.parkreldis.2009.06.007 19632874 PMC2783246

[B67] Qiao Y. Peng D. Jin M. Xue S. (2017). Presenilin 1 Mutation (A431V) causing features of dementia with lewy bodies in a Chinese family of Alzheimer's Disease. J. Alzheimer Dis. Park. 7, 1–4. 10.4172/2161-0460.1000307

[B68] Ratnavalli E. Brayne C. Dawson K. Hodges J. R. (2002). The prevalence of frontotemporal dementia. Neurology 58, 1615–1621. 10.1212/WNL.58.11.1615 12058088

[B69] Roberts A. M. Ware J. S. Herman D. S. Schafer S. Baksi J. Bick A. G.. (2015). Integrated allelic, transcriptional, and phenomic dissection of the cardiac effects of titin truncations in health and disease. Sci. Transl. Med. 7:270ra6. 10.1126/scitranslmed.3010134 25589632 PMC4560092

[B70] Rocca W. A. Bower J. H. Ahlskog J. E. Elbaz A. Grossardt B. R. McDonnell S. K.. (2007). Risk of cognitive impairment or dementia in relatives of patients with Parkinson disease. Arch. Neurol. 64, 1458–1464. 10.1001/archneur.64.10.1458 17923629

[B71] Rovelet-Lecrux A. Deramecourt V. Legallic S. Maurage C. A. Le Ber I. Brice A. (2008). Deletion of the progranulin gene in patients with frontotemporal lobar degeneration or Parkinson disease. Neurobiol. Dis. 31, 41–45. 10.1016/j.nbd.2008.03.004 18479928

[B72] Sannerud R. Esselens C. Ejsmont P. Mattera R. Rochin L. Tharkeshwar A. K.. (2016). Restricted location of PSEN2/γ-secretase determines substrate specificity and generates an intracellular Aβ pool. Cell 166, 193–208. 10.1016/j.cell.2016.05.020 27293189 PMC7439524

[B73] Schulte E. C. Fukumori A. Mollenhauer B. Hor H. Arzberger T. Perneczky R.. (2015). Rare variants in β-Amyloid precursor protein (APP) and Parkinson's disease. Eur. J. Hum. Genet. 23, 1328–1333. 10.1038/ejhg.2014.300 25604855 PMC4592093

[B74] Setó-Salvia N. Pagonabarraga J. Houlden H. Pascual-Sedano B. Dols-Icardo O. Tucci A.. (2012). Glucocerebrosidase mutations confer a greater risk of dementia during Parkinson's disease course. Mov. Disord. 27, 393–399. 10.1002/mds.24045 22173904

[B75] Snider B. J. Norton J. Coats M. Chakraverty S. Hou C. E. Jervis R.. (2005). Novel presenilin 1 mutation (S170F) causing Alzheimer disease with lewy bodies in the third decade of life. Arch. Neurol. 62, 1821–1830. 10.1001/archneur.62.12.1821 16344340

[B76] Srivatsal S. Cholerton B. Leverenz J. B. Wszolek Z. K. Uitti R. J. Dickson D. W.. (2015). Cognitive profile of LRRK2-related Parkinson's disease. Mov. Disord. 30, 728–733. 10.1002/mds.26161 25650144 PMC4397146

[B77] Takao M. Ghetti B. Hayakawa I. Ikeda E. Fukuuchi Y. Miravalle L.. (2002). A novel mutation (G217D) in the Presenilin 1 gene (PSEN1) in a Japanese family: Presenile dementia and parkinsonism are associated with cotton wool plaques in the cortex and striatum. Acta Neuropathol. 104, 155–170. 10.1007/s00401-002-0536-6 12111359

[B78] Tanaka Y. Matsuwaki T. Yamanouchi K. Nishihara M. (2013). Increased lysosomal biogenesis in activated microglia and exacerbated neuronal damage after traumatic brain injury in progranulin-deficient mice. Neuroscience 250, 8–19. 10.1016/j.neuroscience.2013.06.049 23830905

[B79] Terrelonge M. Marder K. S. Weintraub D. Alcalay R. N. (2015). CSF β-amyloid 1-42 predicts progression to cognitive impairment in newly diagnosed Parkinson disease. J. Mol. Neurosci. 58, 88–92. 10.1007/s12031-015-0647-x 26330275 PMC4738011

[B80] Thaler A. Mirelman A. Gurevich T. Simon E. Orr-Urtreger A. Marder K.. (2012). Lower cognitive performance in healthy G2019S LRRK2 mutation carriers. Neurology 79, 1027–1032. 10.1212/WNL.0b013e3182684646 22914834 PMC3430708

[B81] Tomaino C. Bernardi L. Anfossi M. Costanzo A. Ferrise F. Gallo M.. (2007). Presenilin 2 Ser130Leu mutation in a case of late-onset “sporadic” Alzheimer's disease [3]. J. Neurol. 254, 391–393. 10.1007/s00415-006-0373-y 17345043

[B82] Tsuang D. Leverenz J. B. Lopez O. L. Hamilton R. L. Bennett D. A. Schneider J. A.. (2013). APOE epsilon4 increases risk for dementia in pure synucleinopathies. JAMA Neurol. 70, 223–228. 10.1001/jamaneurol.2013.600 23407718 PMC3580799

[B83] Vallania F. L. M. Druley T. E. Ramos E. Wang J. Borecki I. Province M.. (2010). High-throughput discovery of rare insertions and deletions in large cohorts. Genome Res. 20, 1711–1718. 10.1101/gr.109157.110 21041413 PMC2989997

[B84] Van Kampen J. M. Baranowski D. Kay D. G. (2014). Progranulin gene delivery protects dopaminergic neurons in a mouse model of Parkinson's disease. PLoS ONE 9:e97032. 10.1371/journal.pone.0097032 24804730 PMC4013129

[B85] Walker E. S. Martinez M. Brunkan A. L. Goate A. (2005). Presenilin 2 familial Alzheimer's disease mutations result in partial loss of function and dramatic changes in Aβ 42/40 ratios. J. Neurochem. 92, 294–301. 10.1111/j.1471-4159.2004.02858.x 15663477

[B86] Walsh R. Thomson K. L. Ware J. S. Funke B. H. Woodley J. McGuire K. J.. (2017). Reassessment of Mendelian gene pathogenicity using 7,855 cardiomyopathy cases and 60,706 reference samples. Genet. Med. 19, 192–203. 10.1038/gim.2016.90 27532257 PMC5116235

[B87] Weintraub D. Papay K. Siderowf A. (2013). Screening for impulse control symptoms in patients with *de novo* Parkinson disease: a casecontrol study. Neurology 80, 176–180. 10.1212/WNL.0b013e31827b915c 23296128 PMC3589192

[B88] Winder-Rhodes S. E. Evans J. R. Ban M. Mason S. L. Williams-Gray C. H. Foltynie T.. (2013). Glucocerebrosidase mutations influence the natural history of Parkinson's disease in a community-based incident cohort. Brain 136, 392–399. 23413260 10.1093/brain/aws318

[B89] Wright Willis A. Evanoff B. A. Lian M. Criswell S. R. Racette B. A. (2010). Geographic and ethnic variation in Parkinson disease: A population-based study of us medicare beneficiaries. Neuroepidemiology 34, 143–151. 10.1159/000275491 20090375 PMC2865395

[B90] Wu M. C. Lee S. Cai T. Li Y. Boehnke M. Lin X. (2011). Rare-variant association testing for sequencing data with the sequence kernel association test. Am. J. Hum. Genet. 89, 82–93. 10.1016/j.ajhg.2011.05.029 21737059 PMC3135811

