# Supplementary material for: Pleiotropic Effects of Variants in Dementia Genes in Parkinson Disease
Source: Front Neurosci. 2018 Apr 10;12:230. doi: 10.3389/fnins.2018.00230 (PMC5902712; doi:10.3389/fnins.2018.00230)
Supplement: Supplementary file 1 [file Table1.DOCX]

Supplementary Material

**Pleiotropic effects of variants in dementia genes in Parkinson disease**

**Laura Ibanez^1^, Umber Dube^1^, Albert A. Davis^2^, Maria Victoria Fernandez^1^, John Budde^1^, Breanna Cooper^1^, Monica Diez-Fairen^3,4^, Sara Ortega-Cubero^3,5^, Pau Pastor^3,4^, Joel S. Perlmutter^2,6^, Carlos Cruchaga^1¶^, and Bruno A. Benitez^7¶^*.**

*** Correspondence:** Bruno A. Benitez [babenitez@wustl.edu](mailto:babenitez@wustl.edu)

# Supplementary Table 1. Summary of exon coverage per gene in pooled-DNA sequencing

| **Gene** | **Exon** | **Mean Coverage** |
| --- | --- | --- |
| **p53 positive control** |  | 336.0 |
| **pCMV6-XL5 negative control** |  | 111.7 |
| **GRN** | 1 | 158.1 |
|  | 2_4 | 175.3 |
|  | 5_7 | 144.0 |
|  | 8_1 | 205.4 |
|  | 11_13 | 207.9 |
| **PSEN1** | 1 | 221.6 |
|  | 2_3 | 77.8 |
|  | 4 | 80.9 |
|  | 5 | 95.8 |
|  | 6 | 131.5 |
|  | 7 | 154.8 |
|  | 8 | 92.3 |
|  | 9 | 85.5 |
|  | 10 | 156.1 |
|  | 11 | 64.9 |
|  | 12 | 126.0 |
| **PSEN2** | 1_2 | 86.5 |
|  | 3 | 123.3 |
|  | 4 | 140.3 |
|  | 5 | 111.3 |
|  | 6 | 118.9 |
|  | 7_8 | 112.3 |
|  | 9 | 104.7 |
|  | 10_11 | 141.2 |
|  | 12 | 76.1 |
|  | 13 | 74.7 |
| **APP** | 1 | 127.5 |
|  | 2 | 197.2 |
|  | 3 | 145.7 |
|  | 4 | 94.6 |
|  | 5 | 59.5 |
|  | 6 | 130.6 |
|  | 7 | 154.9 |
|  | 8 | 98.9 |
|  | 9 | 123.4 |
|  | 10 | 62.3 |
|  | 11 | 130.4 |
|  | 12 | 124.7 |
|  | 13 | 105.9 |
|  | 14 | 97.8 |
|  | 15 | 184.1 |
|  | 16 | 119.0 |
|  | 17 | 80.4 |
|  | 18 | 64.7 |
