# Supplementary material for: Pleiotropic Effects of Variants in Dementia Genes in Parkinson Disease
Source: Front Neurosci. 2018 Apr 10;12:230. doi: 10.3389/fnins.2018.00230 (PMC5902712; doi:10.3389/fnins.2018.00230)
Supplement: Supplementary file 2 [file Table2.DOCX]

Supplementary Material

**Pleiotropic effects of variants in dementia genes in Parkinson disease**

**Laura Ibanez^1^, Umber Dube^1^, Albert A. Davis^2^, Maria Victoria Fernandez^1^, John Budde^1^, Breanna Cooper^1^, Monica Diez-Fairen^3,4^, Sara Ortega-Cubero^3,5^, Pau Pastor^3,4^, Joel S. Perlmutter^2,6^, Carlos Cruchaga^1¶^, and Bruno A. Benitez^7¶^*.**

*** Correspondence:** Bruno A. Benitez [babenitez@wustl.edu](mailto:babenitez@wustl.edu)

# Supplementary Table 2. Rare variants in the *APP, PSEN1, PSEN2 and GRN* genes in the WUSTL cohort

| **Gene** | **AA Change Annotation^^^** | **Cases**  **(490)** | **MAF^⊥^**  **PD Cases** | **Controls**  **(289)** | **MAF**  **Controls^*^** | **ExAC^φ^** | **MAF**  **ExAC** | **P. Value^#^** | **OR**  **(95% CI)^#^** |
| --- | --- | --- | --- | --- | --- | --- | --- | --- | --- |
| *APP* | S198P  Unknown | 1 | 0.001 | 1 | 0.002 | 49 | 0.0007 | ns | - |
|  | A201V  Non-Pathogenic | 1 | 0.001 | 0 | 0.000 | 10 | 0.0001 | 0.01 | 8.9  (1.1-69.7) |
|  | E599K  Non-Pathogenic | 1 | 0.001 | 1 | 0.002 | 101 | 0.0015 | ns | - |
|  | *BURDEN TEST^⊥^* | 3 | 0.001 | 2 | 0.001 | 59 | 0.0008 | ns | - |
| *GRN* | W7R  Unclear | 1 | 0.001 | 0 | 0.000 | *not found* | - | - | - |
|  | R19W  Non-Pathogenic | 0 | 0.000 | 1 | 0.002 | 16 | 0.0002 | ns | - |
|  | A29V  Novel | 0 | 0.000 | 1 | 0.002 | *not found* | - | - | - |
|  | A324T  Non-Pathogenic | 4 | 0.001 | 1 | 0.002 | 80 | 0.0012 | ns | - |
|  | R433Q  Non-Pathogenic | 3 | 0.003 | 1 | 0.002 | 2 | 0.00003 | 8.25×10^-28^ | 101.8  (16.9-610.6) |
|  | R433W  Non-Pathogenic | 1 | 0.000 | 1 | 0.002 | 239 | 0.0036 | ns | - |
|  | R478H  Unknown | 1 | 0.001 | 0 | 0.000 | 1 | 0.00001 | 1.15×10^-08^ | 67.1  (4.2-1074.6) |
|  | G515A  Unknown | 1 | 0.001 | 0 | 0.000 | 14 | 0.0002 | ns | - |
|  | *BURDEN TEST^⊥^* | 11 | 0.001 | 5 | 0.001 | 352 | 0.0007 | 8.93×10^-03^ | 2.2  (1.2-3.9) |
| *PSEN1* | A79V  Pathogenic | 3 | 0.004 | 0 | 0.000 | 1 | 0.00001 | 3.54×10^-45^ | 268.3  (27.8-2582.4) |
|  | P303L  Novel | 1 | 0.001 | 0 | 0.000 | *not found* | - | - | - |
|  | E318G  Non-Pathogenic | 22 | 0.022 | 10 | 0.020 | 1308 | 0.020 | ns | - |
|  | A360T  Unknown | 3 | 0.003 | 0 | 0.000 | 4 | 0.00006 | 5.22×10^-20^ | 51.0  (11.4-228.4) |
|  | *BURDEN TEST^⊥^* | 7 | 0.003 | 0 | 0.000 | 5 | 0.00002 | 3.16×10^-66^ | 103.6  (32.8-326.0) |
| *PSEN2* | S30F  Unknown | 0 | 0.000 | 1 | 0.002 | 1 | 0.00002 | ns | - |
|  | R62C  Unclear | 0 | 0.000 | 1 | 0.002 | 10 | 0.0002 | ns | - |
|  | R62H  Unclear | 5 | 0.005 | 1 | 0.002 | 236 | 0.0036 | ns | - |
|  | S130K  Unclear | 1 | 0.001 | 0 | 0.000 | 65 | 0.0010 | ns | - |
|  | I154V  Unknown | 1 | 0.001 | 0 | 0.000 | 2 | 0.00003 | 3.89×10^-06^ | 33.9  (3.0-375.0) |
|  | C358R  Novel | 1 | 0.001 | 0 | 0.000 | *not found* | - | - | - |
|  | *BURDEN TEST^⊥^* | 8 | 0.001 | 3 | 0.001 | 314 | 0.00079 | ns | - |

**^φ^** Non-Finnish European Ancestry ExAC individuals

^^^ Annotation according to the AD/FTD Database

^*^ P value for the case-control test did not reach statistically significant values in any variant or gene

^#^ P value, OR and 95% CI corresponding to the Fisher exact test using the WUSTL PD cases and the ExAC non-Finish Europeans as controls

*^⊥^*Burden test includes only variants with MAF<0.01

⊥MAF= Minor allele frequency
