# Supplementary material for: Pleiotropic Effects of Variants in Dementia Genes in Parkinson Disease
Source: Front Neurosci. 2018 Apr 10;12:230. doi: 10.3389/fnins.2018.00230 (PMC5902712; doi:10.3389/fnins.2018.00230)
Supplement: Supplementary file 3 [file Table3.DOCX]

Supplementary Material

**Pleiotropic effects of variants in dementia genes in Parkinson disease**

**Laura Ibanez^1^, Umber Dube^1^, Albert A. Davis^2^, Maria Victoria Fernandez^1^, John Budde^1^, Breanna Cooper^1^, Monica Diez-Fairen^3,4^, Sara Ortega-Cubero^3,5^, Pau Pastor^3,4^, Joel S. Perlmutter^2,6^, Carlos Cruchaga^1¶^, and Bruno A. Benitez^7¶^*.**

*** Correspondence:** Bruno A. Benitez [babenitez@wustl.edu](mailto:babenitez@wustl.edu)

# Supplementary Table 3.

# Table 3A. Summary demographics for carrier of rare variants in the *APP, PSEN1, PSEN2* and *GRN* genes in the WUSTL cohort

**PDD = Parkinson disease with dementia

| **Gene** | **Variant** | **Age at Onset** | ***APOE*** | **Last MMSE** | **Assessment** |
| --- | --- | --- | --- | --- | --- |
| *APP* | A201V | 64 | 33 | 25/30 (2003) | No report of dementia (2003, last follow up) |
|  | E599K | 51 | 33 | 29/30 (2009) | No report of dementia (2009, last follow up) |
|  |  | Control | 33 | 25/30 (1995) | No report of dementia (1995, last follow up) |
|  | S198P | 55 | 33 | 23/30 (2005) | Progressive Dementia, neuropath PDD** |
| *GRN* | A29V | Control | - | - | - |
|  | A324T | 77 | 33 | 16/30 (2004) | Progressive Dementia, potential PDD** |
|  |  | 64 | 33 | 29/30 (2005) | Progressive dementia, neuropath PDD** |
|  |  | 47 | 33 | 28/30 (2016) | No report of dementia (2016, last follow up) |
|  |  | - | - | 14/30 (2005) | Progressive dementia, potential AD |
|  |  | Control | 33 | - | Normal Mental Status (2006, last follow up) |
|  | G515A | 57 | 33 | 29/30 (2012) | No report of dementia (2012, last follow up) |
|  | R19W | Control | 33 | - | - |
|  | R433Q | Control | 33 | - | Normal Mental Status (2008, last follow up) |
|  |  | 38 | 34 | 29/30 (2016) | "Short term memory not as good as it once was" |
|  |  | 42 | 33 | - | No report of dementia (2003, last follow up) |
|  |  | 48 | 33 | - | No report of dementia (1999, last follow up) |
|  | R433W | 59 | 23 | - | No report of dementia (2005, last follow up) |
|  |  | Control | 33 | - | - |
|  | R478H | 63 |  | 30/30 (2015) | No report of dementia (2015, last follow up) |
|  | W7R | 48 | 33 | - | - |
| *PSEN1* | A360T | 43 | 33 | 19/30 (2016) | Dementia |
|  |  | - | 33 | 25/30 (2005) | Dementia |
|  |  | 50 | 34 | 25/30 (2012) | "Occasionally not oriented to time, place, or person" |
|  | A79V | 75 | 34 | - | No report of Dementia (2008, last follow up) |
|  |  | 44 | 33 | - | No report of Dementia (2002, last follow up) |
|  |  | 64 | 23 | 29/30 (2012) | Modified AD8 of 2 in 2012 (self report), reports of delusions (2013) |
|  | E318G | 88 | - | - | "Thinking problems at ~86 years old" |
|  |  | 68 | - | 28/30 (2012) | Early dementia, CDR = 1 (2015 |
|  |  | - | - | - | "Normal Cognition, CDR = 0" (2016) |
|  |  | - | - | 29/30 (2015) | Good memory (2015) |
|  |  | 56 | - | - | "Thinking and memory complaints" (2013) |
|  |  | -- | - | 27/30 (2014) | "Thinking and memory still have remained sharp" (2016 |
|  |  | 56 | - | 28/30 (2014) | "no thinking problems" (2014) |
|  |  | 76 | - | 28/30 (2016) | No report of dementia (2016) |
|  |  | 75 | 34 | 27/30 (2006) | Dementia |
|  |  | 53 | 33 | 28/30 (2007) | No report of Dementia (2007) |
|  |  | Control | 23 | - | - |
|  |  | Control | 33 | - | - |
|  |  | 83 | 34 | 28/30 (1999) | No report of Dementia (2003) |
|  |  | 66 | 33 | 30/30 (2007) | No report of Dementia (2007) |
|  |  | 81 | 33 | 27/30 (2000) | Dementia |
|  |  | 66 | 33 | 19/29 (2013) | Dementia, likely PDD** (2013), Modified AD8 =7 |
|  |  | 67 | 33 | 21/30 (2000) | Dementia, likely AD (2000, not followed up) |
|  |  | 57 | 33 | 30/30 (2012) | No AD on autopsy (2014) |
|  |  | 72 | 33 | 30/30 (1998) | No report of Dementia (2002) |
|  |  | 62 | 34 | 30/30 (2004) | No report of Dementia (2005), "Thinking is good most of the time. She has some difficulty concentrating" (2004 |
|  |  | 50 | 33 | - | "Thinking is good" (2000) |
|  |  | 66 | 33 | 8/30 (2009) | Dementia, possible AD (2000)"Thinking is not good" (2010) ; AD8 = 5 (2010) |
|  |  | - | - | 27/30 (2013) | AD8=2 (2013); "Thinking and memory are good" (2013) |
|  |  | 59 | 33 | 23/30 (2009) | No report of dementia (2011) |
|  |  | 77 | 33 | 25/30 (2003) | Report of Dementia (2003, not followed up) |
|  |  | Control | 33 | - | - |
|  |  | Control | 23 | 30/30 (2007) | - |
|  |  | Control | 33 | - | - |
|  |  | Control | 33 | - | - |
|  |  | Control | 33 | - | - |
|  |  | Control | 33 | - | - |
|  |  | Control | 34 | - | - |
|  | P303L | 45 | - | 27/30 (2014) | No report of Dementia (2015) |
| *PSEN2* | C358R | 48 | 33 | 27/30 (2014) | "short term memory loss" (2015) |
|  | I154V |  | 34 | 25/30 (2014) | "Dementia" (2016) |
|  | R62C | Control | 24 | - | - |
|  | R62H | 65 | 33 | 30/30 (2004) | "problems with concentration" (2004) |
|  |  | Control | 33 | - | - |
|  |  | 59 | - | 15/30 (2016) | Memory Problems (2009) |
|  |  | 52 | - | - | Normal cognition (2011) |
|  |  | 67 | - | 23/30 (2016) | Dementia (2015) |
|  |  | 52 | 33 | 28/30 (2011) | AD on Neuropath (2013) |
|  | S130L | 45 | 33 | 16/30 (2015) | Memory problems (2013) |
|  | S30F | Control | 34 | - | - |

# Table 3B. Summary Demographics for carrier of variants in the *PSEN1 p.A79V and PSEN2 p.S130L* WUSTL cohort

|  | **PSEN1 p.A79V carrier 1** | **PSEN1 p.A79V carrier 2** | **PSEN1 p.A79V carrier 3** | **PSEN2 p.S130L carrier** |
| --- | --- | --- | --- | --- |
| **Age at last clinical assessment** | 86 | 46 | 82 | 66 |
| **Age at onset** | 75 | 44 | 64 | 45 |
| **Disease duration at last assessment (years)** | 11 | 2 | 18 | 21 |
| **Motor symptoms** | Left bradykinesia, left limb and neck rigidity, left rest tremor, and left foot Parkinsonism with Bradykinesia, rigidity, rest tremor, dystonia. | Right bradykinesia, right limb rigidity, right postural tremor and rigidity, bradykinesia, postural tremor, postural instability. | Bilateral lower and upper extremity bradykinesia, more severe on the left, neck rigidity, no rest tremor, postural instability and shuffling gait. | Bilateral lower and upper extremity bradykinesia, neck rigidity, no rest tremor, postural instability, shuffling gait. |
| **MDS-UPDRS III score** | 12/108 | 29/108 | 57/108 | 56/108 |
| **Hoehn & Yahr stage score** | Stage 2 | Stage 2 | Stage 5 | Stage 3 |
| **Years of Levodopa (or equivalent) treatment** | 11 | 2 | 13 | 18 |
| **Available MMSE score** | NA | NA | 29/30 | 16/30 |
| **Depression and medications** | Yes | Yes | Yes | Yes |
| **Visual hallucinations** | No | No | Yes | Yes |
| **Suggestive features REM behavior disorder** | No | No | No | Yes |
