# Supplementary material for: Pleiotropic Effects of Variants in Dementia Genes in Parkinson Disease
Source: Front Neurosci. 2018 Apr 10;12:230. doi: 10.3389/fnins.2018.00230 (PMC5902712; doi:10.3389/fnins.2018.00230)
Supplement: Supplementary file 4 [file Table4.DOCX]

Supplementary Material

**Pleiotropic effects of variants in dementia genes in Parkinson disease**

**Laura Ibanez^1^, Umber Dube^1^, Albert A. Davis^2^, Maria Victoria Fernandez^1^, John Budde^1^, Breanna Cooper^1^, Monica Diez-Fairen^3,4^, Sara Ortega-Cubero^3,5^, Pau Pastor^3,4^, Joel S. Perlmutter^2,6^, Carlos Cruchaga^1¶^, and Bruno A. Benitez^7¶^*.**

*** Correspondence:** Bruno A. Benitez [babenitez@wustl.edu](mailto:babenitez@wustl.edu)

# Supplementary Table 4. Rare variants in the *APP, PSEN1, PSEN2 and GRN* genes in the PPMI cohort

| **Gene** | **AA Change Annotation^^^** | **Cases**  **(331)** | ***MAF***  **PD Cases** | **Controls**  **(134)** | ***MAF***  **Controls^*^** | **ExAC^φ^** | ***MAF***  **ExAC** | **P. Value^#^** | **OR**  **(95% CI)^#^** |
| --- | --- | --- | --- | --- | --- | --- | --- | --- | --- |
| APP | Q138R  Novel | 0 | 0.000 | 1 | 0.004 | *not found* | - | - | - |
|  | S198P  Unknown | 0 | 0.000 | 1 | 0.004 | 49 | 0.0007 | ns | - |
|  | R398T  Novel | 1 | 0.002 | 0 | 0.000 | *not found* | - | - | - |
|  | A479S  Unknown | 0 | 0.000 | 1 | 0.004 | 7 | 0.0001 | ns | - |
|  | R499C  Novel | 1 | 0.002 | 0 | 0.000 | *not found* | - | - | - |
|  | *BURDEN TEST^⊥^* | 2 | <0.001 | 3 | 0.002 | 56 | 0.0002 | ns | - |
| GRN | D108N  Non-Pathogenic | 1 | 0.002 | 0 | 0.000 | *not found* | *-* | ns | - |
|  | S120Y  Non-Pathogenic | 0 | 0.000 | 1 | 0.004 | 32 | 0.005 | ns | - |
|  | C260R  Novel | 0 | 0.000 | 1 | 0.004 | *not found* | - | - | - |
|  | A324T  Non-Pathogenic | 1 | 0.002 | 0 | 0.000 | 80 | 0.0012 | ns | - |
|  | R433W  Non-Pathogenic | 5 | 0.008 | 0 | 0.000 | 239 | 0.0005 | ns | - |
|  | *BURDEN TEST^⊥^* | 7 | 0.002 | 2 | 0.002 | 351 | 0.001 | ns | - |
| PSEN1 | R220Q  Unknown | 1 | 0.002 | 0 | 0.000 | 1 | 0.00001 | 2.15×10^-12^ | 100.8  (6.2-1613.4) |
|  | E318G  Non-Pathogenic | 9 | 0.010 | 3 | 0.010 | 1308 | 0.020 | ns | - |
|  | *BURDEN TEST^⊥^* | 1 | 0.002 | 0 | 0.000 | 1 | 0.00001 | ns | - |
| PSEN2 | G56S  Unknown | 1 | 0.002 | 0 | 0.000 | 7 | 0.00011 | 9.43×10^-04^ | 14.2  (1.7-117.6) |
|  | R62H  Unclear | 2 | 0.003 | 0 | 0.000 | 236 | 0.0036 | ns | - |
|  | R71W  Unclear | 2 | 0.003 | 5 | 0.019^+^ | 248 | 0.0037 | ns | - |
|  | S130L  Unclear | 1 | 0.002 | 1 | 0.004 | 65 | 0.00098 | ns | - |
|  | K161R  Unclear | 1 | 0.002 | 0 | 0.000 | *not found* | - | - | - |
|  | V393M  Unclear | 0 | 0.000 | 1 | 0.004 | 16 | 0.00024 | ns | - |
|  | *BURDEN TEST^⊥^* | 7 | 0.002 | 7 | 0.004 | 572 | 0.0014 | ns | - |

**^φ^** Non-Finnish European Ancestry ExAC individuals

^^^ Annotation according to the AD/FTD Database

^*^ P value for the case-control test did not reach statistically significant values in any variant or gene

^#^ P value, OR and 95% CI corresponding to the Fisher exact test using the PPMI PD cases and the ExAC non-Finish Europeans as controls

*^⊥^* Burden test includes only variants with *MAF*<0.01

^+^ The Fisher exact test corresponding to the case-control analysis was nominally significant (p=0.01; OR=0.16 (0.02-0.98))
