# Supplementary material for: Pleiotropic Effects of Variants in Dementia Genes in Parkinson Disease
Source: Front Neurosci. 2018 Apr 10;12:230. doi: 10.3389/fnins.2018.00230 (PMC5902712; doi:10.3389/fnins.2018.00230)
Supplement: Supplementary file 5 [file Table5.DOCX]

Supplementary Material

**Pleiotropic effects of variants in dementia genes in Parkinson disease**

**Laura Ibanez^1^, Umber Dube^1^, Albert A. Davis^2^, Maria Victoria Fernandez^1^, John Budde^1^, Breanna Cooper^1^, Monica Diez-Fairen^3,4^, Sara Ortega-Cubero^3,5^, Pau Pastor^3,4^, Joel S. Perlmutter^2,6^, Carlos Cruchaga^1¶^, and Bruno A. Benitez^7¶^*.**

*** Correspondence:** Bruno A. Benitez [babenitez@wustl.edu](mailto:babenitez@wustl.edu)

# Supplementary Table 5. Summary Demographics for carrier of rare variants in the *APP, PSEN1, PSEN2 and GRN* genes in the PPMI cohort

| **Gene** | **Variant** | **Age at Onset** | **MoCA** | **APOE** |
| --- | --- | --- | --- | --- |
| APP | R397T | 56 | 23 | 34 |
|  | A479S | 69 | 28 | 33 |
|  | Q138R | 82 | 28 | 33 |
|  | R499C | 62 | 30 | - |
|  | S198P | 61 | 28 | 33 |
| GRN | A324T | 71 | 23 | 33 |
|  | C260R | 79 | 27 | 33 |
|  | D108N | 75 | 28 | 33 |
|  | R433W | 75 | 25 | 33 |
|  |  | 58 | 27 | 33 |
|  |  | 58 | 27 | 33 |
|  |  | 71 | 28 | 33 |
|  |  | 85 | 21 | - |
|  | S120Y | 61 | 27 | 34 |
| PSEN1 | E318G | 80 | 27 | 33 |
|  |  | 58 | 27 | 33 |
|  |  | 57 | 28 | 34 |
|  |  | 67 | 29 | 23 |
|  |  | 75 | 27 | 33 |
|  |  | 39 | 29 | 33 |
|  |  | 50 | 30 | 33 |
|  |  | 57 | 23 | 34 |
|  |  | 45 | 30 | 34 |
|  |  | 62 | 30 | 34 |
|  |  | 43 | 30 | 34 |
|  |  | 43 | 29 | 44 |
|  | R220Q | 47 | 26 | 33 |
| PSEN2 | G56S | 39 | 29 | 33 |
|  | K161R | 65 | 29 | 33 |
|  | R62H | 72 | 23 | 34 |
|  | S130L | 47 | 30 | 33 |
|  |  | 66 | 27 | 34 |
|  |  | 65 | 29 | 33 |
|  | V393M | 70 | 28 | 33 |
