# Supplementary material for: Pleiotropic Effects of Variants in Dementia Genes in Parkinson Disease
Source: Front Neurosci. 2018 Apr 10;12:230. doi: 10.3389/fnins.2018.00230 (PMC5902712; doi:10.3389/fnins.2018.00230)
Supplement: Supplementary file 6 [file Table6.DOCX]

Supplementary Material

**Pleiotropic effects of variants in dementia genes in Parkinson disease**

**Laura Ibanez^1^, Umber Dube^1^, Albert A. Davis^2^, Maria Victoria Fernandez^1^, John Budde^1^, Breanna Cooper^1^, Monica Diez-Fairen^3,4^, Sara Ortega-Cubero^3,5^, Pau Pastor^3,4^, Joel S. Perlmutter^2,6^, Carlos Cruchaga^1¶^, and Bruno A. Benitez^7¶^*.**

*** Correspondence:** Bruno A. Benitez [babenitez@wustl.edu](mailto:babenitez@wustl.edu)

# Supplementary Table 6. Rare variants in the *APP, PSEN1, PSEN2 and GRN* genes in the UN cohort

| **Gene** | **AA Change Annotation^^^** | **Cases**  **(553)** | **MAF**  **PD Cases** | **Controls (550)** | **MAF**  **Controls** | **P. Value^#^** | **OR**  **(95% CI)^#^** |
| --- | --- | --- | --- | --- | --- | --- | --- |
| APP | E559K  Non-Pathogenic | 2 | 0.002 |  |  |  |  |
|  | *BURDEN TEST^⊥°^* | - | - |  |  |  |  |
| GRN | R212Q  Non-Pathogenic | 1 | 0.001 |  |  |  |  |
|  | A324T  Non-Pathogenic | 2 | 0.002 |  |  |  |  |
|  | R433W  Non-Pathogenic | 2 | 0.002 |  |  |  |  |
|  | G515A  Unknown | 1 | 0.001 |  |  |  |  |
|  | *BURDEN TEST^⊥^* | 6 | 0.001 |  |  |  |  |
| PSEN1 | E318G  Non-Pathogenic | 20 | 0.02 | 14 | 0.01 | ns |  |
|  | T354I  Unknown | 1 | 0.001 | 0 | *0* |  |  |
|  | A360T  Unclear | 1 | 0.001 | 0 | 0 |  |  |
|  | *BURDEN TEST^⊥°^* | 22 | 0.001 | 14 |  | ns |  |
| PSEN2 | R62H  Unclear | 9 | 0.007 |  |  |  |  |
|  | S130L  Unclear | 7 | 0.006 |  |  |  |  |
|  | V148I  Pathogenic | 1 | 0.001 | 0 | 0 | ns |  |
|  | *BURDEN TEST^⊥^* | 16 | 0.016 |  |  |  |  |

^ Annotation according to the AD/FTD Database

*^⊥^* Burden test includes only variants with MAF<0.01

° Burden test not performed due to the inclusion of only one variant
