# Supplementary material for: Pleiotropic Effects of Variants in Dementia Genes in Parkinson Disease
Source: Front Neurosci. 2018 Apr 10;12:230. doi: 10.3389/fnins.2018.00230 (PMC5902712; doi:10.3389/fnins.2018.00230)
Supplement: Supplementary file 7 [file Table7.DOCX]

Supplementary Material

**Pleiotropic effects of variants in dementia genes in Parkinson disease**

**Laura Ibanez^1^, Umber Dube^1^, Albert A. Davis^2^, Maria Victoria Fernandez^1^, John Budde^1^, Breanna Cooper^1^, Monica Diez-Fairen^3,4^, Sara Ortega-Cubero^3,5^, Pau Pastor^3,4^, Joel S. Perlmutter^2,6^, Carlos Cruchaga^1¶^, and Bruno A. Benitez^7¶^*.**

*** Correspondence:** Bruno A. Benitez [babenitez@wustl.edu](mailto:babenitez@wustl.edu)

# Supplementary Table 7. Summary Demographics for carrier of rare variants in the *APP, PSEN1, PSEN2 and GRN* genes in the UN cohort.

| **Gene** | **Variant** | **Age at onset** | **APOE** | **Family History§** | **Parkinsonism** | **Disease duration*** | **Dementia^¶^** | **tUDPRS** | **Psychiatric comorbidities** |
| --- | --- | --- | --- | --- | --- | --- | --- | --- | --- |
| GRN | A324T | 63 |  | No | Classic | 1 | No | 2 | No |
|  |  | 72 | 33 | Positive | Akinetic-rigid | 2 | No | 11 | No |
|  | *R212Q* | *71* | 33 | Positive | Classic | 3 | No | 16 | No |
|  | R433W | 53 | 33 | No | Classic | 12 | No | 30 | Impulse disorders |
|  |  | 62 |  | No | Classic | 8 | No | 16 | Depression, Hallucinations |
| PSEN1 | A360T | 72 | 33 | No | Tremor-dominant | 6 | Yes | 18 | No |
|  |  | *46* |  | No | Classic | 11 | No | 9 | No |
|  | E318G | 62 | 33 | No | Classic | 8 | Yes | 18 | No |
|  |  | 68 | 34 | Unknown | Akinetic-rigid | 7 | yes | 0 | Hallucinations |
|  |  | 32 | 34 | Positive | Classic | 6 | No | 19 | No |
|  |  | 48 | 23 | No | Classic | 11 | No | 7 | No |
|  |  | 59 | 33 | No | Classic | 12 | yes | 19 | Hallucinations |
|  |  | 62 | 34 | No | Classic | 9 | No | 15 | No |
|  |  | 54 | 23 | No | Classic | 16 | No | 5 | Depression |
|  |  | 65 | 33 | Unknown | Classic | 12 | No | 16 | No |
|  |  | 59 | 22 | No | Akinetic-rigid | 18 | No | 7 | Hallucinations |
|  |  | 72 |  | No | Classic | 9 | No | 16 | No |
|  |  | 66 | 33 | No | Classic | 5 | Yes | 29 | Depression, Hallucinations |
|  |  | 44 | 33 | No | Classic | 3 | No | 14 | No |
|  |  | 49 |  | No | Classic | 7 | No | 10 | No |
|  |  | 68 | 33 | No | Akinetic-rigid | 1 | No | 6 | No |
|  |  | 55 | 34 | No | Classic | 15 | No | 20 | No |
|  |  | 64 | 33 | No | Classic | 1 | No | 0 | Bipolar disorder |
|  |  | 70 | 23 | No | Akinetic-rigid | 3 | Yes | 21 | Anxiety |
|  |  | 83 | 34 | No | unknown | 0 | 0 | 0 | No |
|  |  | 59 | 34 | Positive | Classic | 4 | No | 23 | No |
|  |  | 73 | 33 | No | Classic | 4 | Yes | 5 | No |
|  |  | 78 |  | No | Akinetic-rigid | 7 | No | 11 | No |
|  |  | 80 | 33 | No | unknown | 0 | 0 | 0 | No |
|  |  | 59 | 33 | No | Classic | 9 | No | 8 | No |
|  |  | 63 | 33 | No | Tremor-dominant | 2 | No | 13 | No |
|  |  | 46 |  | Unknown | Akinetic-rigid | 13 | No | 19 | No |
|  |  | *72* |  | Positive | Classic | 3 | Yes | 28 | No |
|  |  | *55* | 33 | No | Classic | 3 | No | 13 | No |
|  | T354I | 73 | 34 | Unknown | Classic | 5 | Yes | 25 | Irritability, Hallucinations |
|  |  | *57* |  | No | Classic | 12 | No | 10 | No |
| PSEN2 | R62H | 71 | 33 | No | Akinetic-rigid | 5 | No | 8 | No |
|  |  | 55 | 33 | No | Tremor-dominant | 5 | No | 22 | No |
|  |  | 62 | 23 | Positive | Classic | 7 | No | 20 | Depression |
|  |  | 19 | 33 | No | Classic | 10 | No | 7 | Hyperactivity, euphoria |
|  |  | 60 | 34 | No | Classic | 5 | No | 17 | No |
|  |  | 72 | 33 | No | Akinetic-rigid | 9 | No | 11 | No |
|  |  | 69 |  | Positive | unknown | 0 | 0 | 0 | No |
|  |  | 44 |  | Unknown | Classic | 17 | No | 4 | No |
|  |  | 59 | 33 | Unknown | Tremor-dominant | 3 | No | 9 | Depression |
|  | S130L | 69 | 33 | No | Classic | 9 | No | 22 | Depression, Hallucinations |
|  |  | 76 | 33 | No | Classic | 7 | No | 20 | Anxiety |
|  |  | 64 | 33 | Positive | Tremor-dominant | 4 | No | 8 | No |
|  |  | 66 | 33 | No | Classic | 5 | Yes | 14 | Delirium |
|  |  | 49 | 33 | Positive | Classic | 15 | Yes | 14 | Hallucinations |
|  |  | 59 | 33 | No | Tremor-dominant | 9 | No | 8 | No |
|  |  | 55 |  | Unknown | unknown | 0 | 0 | 0 | No |
|  | V148I | 25 |  | Positive | Tremor-dominant | 12 | No | 13 | No |

**§** PD subjects with at least one affected first-degree relative were classified as familial PD (Positive)

***** Disease duration in years

**^¶^** Diagnosis of dementia was done when cognitive impairment was ≥2 following item 3 of the UPDRS I scale, cognitive impairment interfered with daily living activities and Mini-Mental State Examination (MMSE) score <24.

tUDPRS=total UPDRS-III score
