# Supplementary material for: Pleiotropic Effects of Variants in Dementia Genes in Parkinson Disease
Source: Front Neurosci. 2018 Apr 10;12:230. doi: 10.3389/fnins.2018.00230 (PMC5902712; doi:10.3389/fnins.2018.00230)
Supplement: Supplementary file 8 [file Table8.DOCX]

Supplementary Material

**Pleiotropic effects of variants in dementia genes in Parkinson disease**

**Laura Ibanez^1^, Umber Dube^1^, Albert A. Davis^2^, Maria Victoria Fernandez^1^, John Budde^1^, Breanna Cooper^1^, Monica Diez-Fairen^3,4^, Sara Ortega-Cubero^3,5^, Pau Pastor^3,4^, Joel S. Perlmutter^2,6^, Carlos Cruchaga^1¶^, and Bruno A. Benitez^7¶^*.**

*** Correspondence:** Bruno A. Benitez [babenitez@wustl.edu](mailto:babenitez@wustl.edu)

# Supplementary Table 8. *APOE* genotypes and allelic frequencies in PD cases and controls

**A. *APOE* genotypes and allelic frequencies in PD cases and controls in WUSTL and PPMI cohorts**

| **Cohort** | **WUSTL** | | | | **PPMI** | | | **Joint Analyses** | | |
| --- | --- | --- | --- | --- | --- | --- | --- | --- | --- | --- |
| **ApoE Genotype** | **Cases**  **(448)** | **Controls**  **(313)** | **p. Value** | **Cases**  **(316)** | | **Controls**  **(130)** | **p. Value** | **Cases**  **(764)** | **Controls**  **(443)** | **p. Value** |
| **2/2** | <0.01 | 0.01 | ns | 0.01 | | 0.02 | ns | <0.01 | 0.01 | ns |
| **2/3** | 0.10 | 0.12 | ns | 0.15 | | 0.08 | ns | 0.12 | 0.11 | ns |
| **2/4** | 0.02 | 0.04 | ns | 0.03 | | 0.04 | ns | 0.02 | 0.04 | ns |
| **3/3** | 0.61 | 0.58 | ns | 0.61 | | 0.65 | ns | 0.61 | 0.60 | ns |
| **3/4** | 0.25 | 0.23 | ns | 0.19 | | 0.19 | ns | 0.22 | 0.22 | ns |
| **4/4** | 0.01 | 0.02 | ns | 0.02 | | 0.02 | ns | 0.02 | 0.02 | ns |
| **ApoE Allele Frequency** |  |  |  |  | |  |  |  |  |  |
| **2** | 0.07 | 0.09 | ns | 0.09 | | 0.07 | ns | 0.08 | 0.09 | ns |
| **3** | 0.78 | 0.76 | ns | 0.78 | | 0.79 | ns | 0.78 | 0.77 | ns |
| **4** | 0.15 | 0.15 | ns | 0.13 | | 0.14 | ns | 0.14 | 0.15 | ns |

**B. *APOE* genotypes and allelic frequencies in PD cases and controls in UN cohorts**

| **Cohort** | **UN** | | |
| --- | --- | --- | --- |
| **ApoE Genotype** | **Cases** | **Controls** | **p. Value** |
|  | **528** | **640** |  |
| **2/2** | 0.004 | 0.003 | ns |
| **2/3** | 0.089 | 0.091 | ns |
| **2/4** | 0.011 | 0.011 | ns |
| **3/3** | 0.752 | 0.722 | ns |
| **3/4** | 0.138 | 0.164 | ns |
| **4/4** | 0.006 | 0.009 | ns |
| **ApoE Allele Frequency** |  |  |  |
| **2** | 0.05 | 0.05 | ns |
| **3** | 0.87 | 0.87 | ns |
| **4** | 0.08 | 0.10 | ns |
